# Decoding organ fibrosis: mechanistic insights and emerging therapeutic strategies

**DOI:** 10.1038/s41392-025-02532-0

**Published:** 2026-03-06

**Authors:** Xiangqi Chen, Jinhang Zhang, Ling Guo, Chuan Wu, Jingyue Zhou, Mingzhu Xu, Li Mo, Yanping Li, Jinhan He

**Affiliations:** 1 Department of Pharmacy, Institute of Metabolic Diseases and Pharmacotherapy, West China Hospital, Sichuan University, Chengdu, China; 2 National Engineering Technology Research Center for Miao Medicine, Guizhou Engineering Technology Research Center for Processing and Preparation of Traditional Chinese Medicine and Ethnic Medicine, College of Pharmaceutical Sciences, Guizhou University of Traditional Chinese Medicine, Guiyang, China; 3 State Key Laboratory of Biotherapy, West China Hospital, Sichuan University, Chengdu, China; 4 West China School of Pharmacy, Sichuan University, Chengdu, China; 5 Center of Gerontology and Geriatrics, West China Hospital, Sichuan University, Chengdu, China

**Keywords:** Inflammation, Drug development, Health care

## Abstract

Fibrosis is a maladaptive pathophysiological process characterized by excessive deposition of extracellular matrix resulting from dysregulated tissue repair responses. Fibrosis can affect nearly all organ systems, such as the lung, heart, liver, and kidney. Persistent fibrotic remodeling leads to architectural distortion, loss of function, organ failure, and ultimately increased mortality. These devastating outcomes highlight the urgent need for effective antifibrotic therapies. Advances in multiomics technologies have revealed that fibrosis represents a dynamic alteration spanning the molecular, cellular, microenvironmental, and organ levels. Despite impressive progress in our understanding of fibrogenesis over recent years, a substantial translational gap remains between identifying potential antifibrotic targets and translating this theoretical knowledge into effective human therapies. To further understand pathogenesis and facilitate the development of novel antifibrotic drugs, this review summarizes crucial milestones in fibrosis research, elaborates on organ-specific pathogenic mechanisms, and details the phenotypic and functional changes in critical cellular players, including parenchymal cells, fibroblasts, endothelial cells, and immune cells. Furthermore, this review outlines the key signaling pathways implicated in the pathogenesis of fibrosis, provides a comprehensive overview of relevant clinical trials, and discusses promising future research directions, including cross-organ multiomics integration, chimeric antigen receptor therapy, and artificial intelligence technology applications.

## Introduction

Fibrosis is a chronic and progressive pathophysiological response to tissue injury. Under normal conditions, appropriate collagen deposition is essential for maintaining tissue integrity and facilitating repair. However, severe or repetitive injury disrupts the delicate balance between extracellular matrix (ECM) synthesis and degradation, leading to excessive accumulation of collagen, fibronectin, and other ECM components.^1^ This pathological ECM deposition, termed fibrosis or scarring, progressively impairs organ architecture and function, ultimately culminating in organ failure and increased mortality. Common fibrosis-related diseases include metabolic dysfunction-associated steatotic liver disease (MASLD), chronic kidney disease (CKD), myocardial infarction, heart failure, idiopathic pulmonary fibrosis (IPF), and scleroderma, which affect nearly every organ system.^2^ With an annualized incidence of major fibrosis-related conditions near 5%, fibrosis has become a pervasive global health crisis, affecting an estimated 25% of the world’s population.^3^ To date, the FDA has approved only two direct antifibrotic drugs—pirfenidone and nintedanib—for IPF, and resmetirom and semaglutide (both act as indirect modulators) for metabolic dysfunction-associated steatohepatitis (MASH).^4,5^ However, these treatments merely slow disease progression rather than reversing established fibrosis in the liver or lung. Thus, there is an urgent need to deepen our understanding of the pathogenesis of organ fibrosis, identify potential therapeutic targets, and alleviate bottlenecks in drug development.

**Fig. 1:**
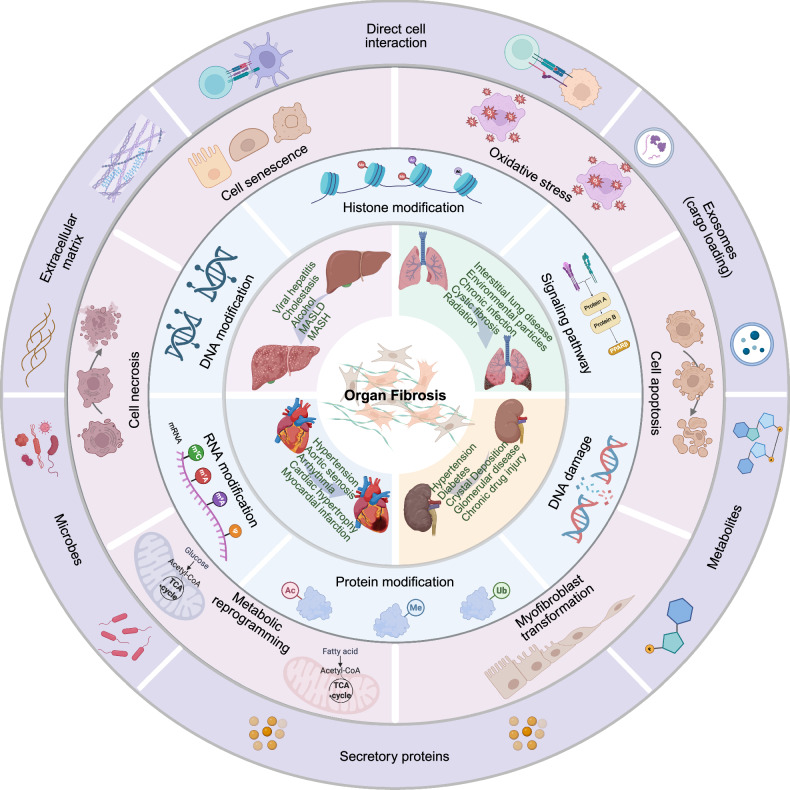
Overview of the etiologies and mechanisms of organ fibrosis. Normal organs can develop fibrosis when exposed to various causative factors, such as environmental particles, chronic infections, radiation, hypertension, and diabetes mellitus. In response to injury, cellular components undergo changes such as signaling pathway activation, epigenetic remodeling, and DNA damage. These changes lead to cellular events such as apoptosis, necrosis, metabolic reprogramming, senescence, and epithelial‒mesenchymal transition (EMT), which subsequently alter the tissue microenvironment, including intercellular interactions and the production of profibrotic effectors (e.g., cytokines, metabolites, and extracellular matrix components), thus regulating organ fibrogenesis. This tool was created with BioRender (https://www.biorender.com/)

Excessive activation of ECM-producing cells is central to fibrosis, with fibroblasts and myofibroblasts recognized as primary contributors. However, emerging evidence implicates epithelial cells, endothelial cells, and bone marrow-derived cells (e.g., fibrocytes and macrophages) as potential myofibroblast precursors. Advances in multiomics have revealed substantial spatiotemporal heterogeneity in parenchymal, stromal, and immune cells, confirming that fibrosis is a highly complex and multicellular process.^6^ The fibrogenic niche comprises not only ECM-producing cells but also parenchymal cells, vascular endothelial cells, immune subsets (macrophages, dendritic cells, granulocytes, T cells, and B cells), and a dynamic milieu of profibrotic/antifibrotic mediators (e.g., cytokines, metabolites, and extracellular vesicles) embedded within a remodeled ECM.^7^ Critical determinants steering tissue toward fibrosis or regeneration include molecular alterations (e.g., epigenetic modifications, DNA damage), cellular events (e.g., apoptosis/necrosis, senescence, metabolic reprogramming), and altered intercellular communication driven by shifting effector molecules and ECM components (Fig. 1).^4^ These insights underscore the need to move beyond a fibroblast-centric view and embrace a holistic, systems-level understanding of fibrosis pathogenesis.

In this review, we first outline key milestones in fibrosis research and then delve into the epidemiology, etiology, and disease-specific mechanisms underlying fibrosis in the liver, lung, heart, and kidney, while highlighting shared critical signaling pathways. We also summarize recent clinical trials for fibrotic disorders and discuss future directions, including cross-organ multiomics integration, chimeric antigen receptor (CAR) therapy, and the application of artificial intelligence (AI) in fibrosis research and drug discovery.

## Historical discoveries and milestone events in organ fibrosis

**Fig. 2:**
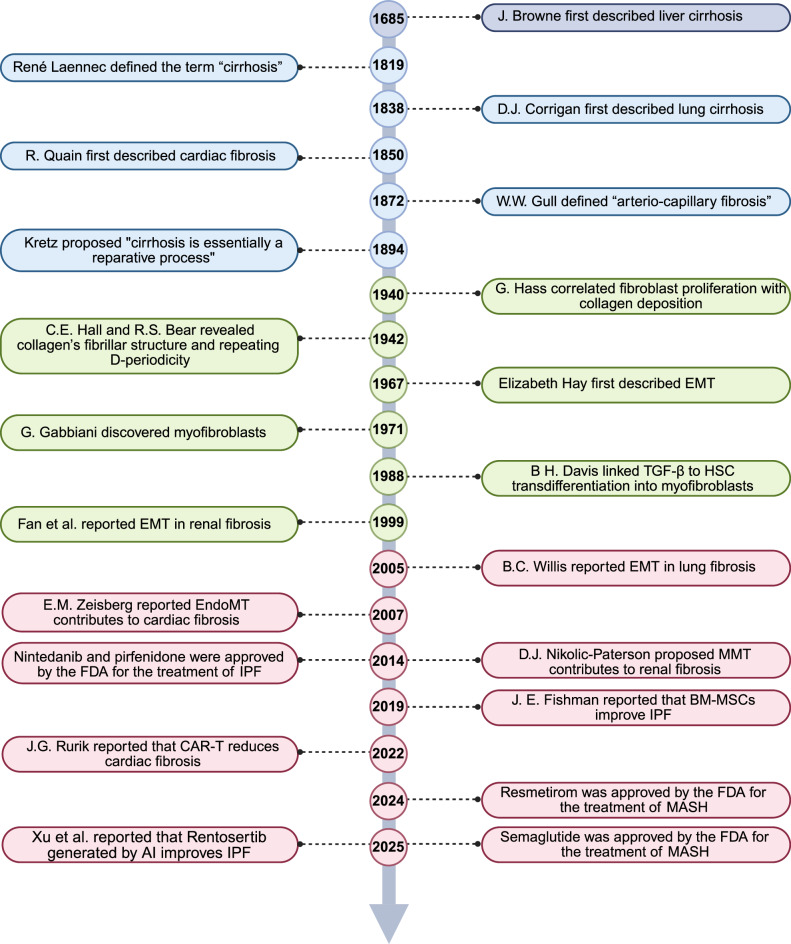
Timeline of the milestones in organ fibrosis. EMT epithelial-to-mesenchymal transformation, TGF-β transforming growth factor-β, HSC hepatic stellate cell, EndoMT endothelial-to-mesenchymal transformation, MMT macrophage-to-mesenchymal transition, IPF idiopathic pulmonary fibrosis, BM-MSCs bone marrow-derived mesenchymal stem cells, CAR-T chimeric antigen receptor T cells, MASH metabolic dysfunction-associated steatohepatitis, AI artificial intelligence. This tool was created with BioRender (https://www.biorender.com/)

The understanding of fibrosis originated from postmortem examinations, where abnormal structural changes in organs were first documented. In 1685, pathologist J. Browne provided the earliest known description of liver cirrhosis, characterizing it as “a glandulous-appearing liver” (Fig. 2).^8^ In 1819, René Laennec formally introduced the term “cirrhosis”, derived from the Greek word κίρρωση (kirrhosi), meaning tawny or orange‒brown.^9^ By 1838, R. Carswell further refined the histological definition, describing liver cirrhosis as “a state of atrophy with variable amounts and distribution of contractile fibrous tissue, cirrhotic nodules, and vascular compression”.^8^ Concurrently, D.J. Corrigan documented pulmonary fibrosis, noting “a fibrous covering, a great quantity of strong cellular tissue, and the elastic lining of the lung tubes”.^10^ Cardiac fibrosis was first identified in 1850 by R. Quain as “a fatty disease of the heart characterized by the presence of peculiar fatty matter within the heart’s texture”.^11^ In 1872, W.W. Gull made a pivotal observation in renal pathology, identifying a fine hyalin-fibroid substance present between the convoluted tubules, which caused the tubules to appear wider than normal, a condition he termed “arterio-capillary fibrosis”.^12^

Despite these foundational descriptions, 19th-century pathologists were limited by their reliance on autopsy specimens, which revealed only end-stage fibrotic changes. A critical advancement came with the advent of experimental animal models, enabling researchers to study fibrosis dynamically. In 1894, Kretz proposed that “cirrhosis is essentially a reparative process”, a theory substantiated in 1906 by R. M. Pearce’s experiments in dogs.^13^ Pearce induced hepatic necrosis via hemolytic immune serum and meticulously documented the ensuing repair process, including early hepatic cell karyokinesis, endothelial cell proliferation, granulation tissue formation, and progressive connective tissue fibrosis leading to cirrhosis. Similarly, in 1938, J.B. Duguid’s study on rat nephritis induced by acid sodium phosphate and calciferol revealed renal fibrosis as a consequence of interstitial collapse and aberrant connective tissue deposition.^14^ These studies underscore fibrosis as a maladaptive remodeling process, although the origins and specific properties of fibrotic tissue remain unclear.

The 1930s marked the emergence of ECM research, with collagen identified as its primary structural component.^15^ By 1942, C.E. Hall and R.S. Bear revealed collagen’s fibrillar structure and repeated D-periodicity via electron microscopy and X-ray diffraction, respectively.^16^ Between 1950 and 1975, distinct collagen types (I, II, III) were identified,^15^ paving the way for organ-specific fibrosis studies. In 1979, M. Rojkind demonstrated that cirrhotic livers exhibited a disproportionate increase in type I over type III collagen. In 1981, E.D. Bateman localized type I collagen in pulmonary fibrosis scars, whereas in 1989, K. Yoshika linked type III collagen to glomerular pathology in chronic kidney disease. That same year, K.T. Weber elucidated the role of fibrillar collagen (types I and III) in myocardial fibrosis, revealing how pressure overload induces pathological ECM remodeling.^17^

The cellular basis of fibrosis began to unfold in 1940, when G. Hass correlated fibroblast proliferation with collagen deposition and degradation.^18^ A breakthrough came in 1971 with G. Gabbiani’s discovery of myofibroblasts, hybrid cells exhibiting both synthetic and contractile functions in granulation tissue.^19^ Later studies revealed that persistent and excessive myofibroblast activation drives pathological fibrosis. The pivotal role of transforming growth factor (TGF) was established in 1980 when A.B. Roberts isolated it from transformed cells and demonstrated its fibrogenic effects.^20^ By 1988, B.H. Davis linked TGF-β to hepatic stellate cell (HSC) transdifferentiation into myofibroblasts in hepatic fibrosis.^21^ Research has subsequently explored myofibroblast origins, including epithelial‒mesenchymal transition (EMT), which was first described during embryogenesis by Elizabeth Hay in 1967.^22^ In 1999, Fan et al. demonstrated that TGF-β induced EMT in renal fibrosis,^23^ whereas B.C. Willis extended this concept to pulmonary fibrosis in 2005.^24^ EMT is characterized by the loss of epithelial markers (e.g., E-cadherin, cytokeratins, and occludins) and the acquisition of mesenchymal markers (e.g., N-cadherin, vimentin, fibronectin, and α-SMA) and is now recognized as a fundamental pathogenic process in organ fibrosis. Endothelial–mesenchymal transition (EndoMT), a form of EMT that takes place throughout the heart’s embryonic development, was linked to cardiac fibrosis in 2007 by E.M. Zeisberg.^25^ In 2014, D.J. Nikolic-Paterson described macrophage-to-mesenchymal transition (MMT) as a source of renal myofibroblasts.^26^

In recent decades, significant strides have been made in antifibrotic therapies. Pirfenidone (Esbriet®, Roche Registration GmbH) was approved for the treatment of IPF in Europe in 2011 and in the USA in 2014.^27^ Nintedanib (Ofev®, Boehringer Ingelheim International GmbH), a multikinase inhibitor, received its first approval in the USA in 2014 for the treatment of IPF.^28^ Cell-based therapies, such as J. E. Fishman’s 2019 trial using bone marrow-derived mesenchymal stem cells (BM-MSCs) to slow lung fibrosis,^29^ and J.G. Rurik’s 2022 chimeric antigen receptor T-cell (CAR-T) approach for cardiac fibrosis,^30^ highlight regenerative potential. In 2024, Resmetirom (MGL-3196), a first-in-class selective THR-β agonist approved by the FDA for the treatment of noncirrhotic MASH with moderate to advanced fibrosis, represents a watershed moment in MASLD therapy.^4^ Cutting-edge tools such as AI are accelerating drug discovery. In 2025, Xu et al. reported promising phase II results for Rentosertib, a first-in-class AI-generated kinase inhibitor for IPF.^31^ Today, single-cell RNA sequencing (scRNA-seq), spatial transcriptomics, and gene editing are revealing fibrosis at unprecedented resolution. These advances, combined with AI and precision medicine, herald a new era of targeted therapies for fibrotic diseases across various organs.

## Epidemiology, etiology, and pathophysiology of organ-specific fibrosis

Organ fibrosis, mainly including hepatic, pulmonary, cardiac, and renal fibrosis, is widely recognized as the common outcome of organ injury and is characterized by excessive ECM deposition.^2^ The fibrotic cascade initiates parenchymal damage, triggering inflammatory cascades that subsequently activate resident fibroblasts and promote the formation of a profibrotic niche. Sustained ECM accumulation resulting from persistent injury disrupts tissue architecture and compromises organ function, ultimately progressing to end-stage organ failure.^32^ The underlying pathogenesis involves not only cell-autonomous signaling pathways but also complex intercellular communication networks within the fibrotic microenvironment. While these disorders share common pathological features, emerging evidence from different fibrotic models reveals substantial organotypic variation in the cellular effectors and molecular mechanisms driving fibrogenesis (Table 1). Recent advances in single-cell omics technologies have enabled the identification of previously unrecognized cellular subpopulations that contribute to tissue-specific fibrotic responses (Table 2). These findings underscore the necessity for systematic investigation of (1) principal effector cell populations, (2) their functionally distinct subclusters, and (3) the unique intercellular crosstalk that orchestrates organ-specific fibrogenesis.

**Table 1:** Animal models relevant to organ fibrosis

Organ	Induce method	Pathological/characteristics	Duration	Advantages	Limitations	Reference
Liver	CCl₄-induced	Hepatocyte injury, inflammation, and ECM production, Serum ALT/AST increased	4–12 weeks	Simplicity; high reproducibility; low cost; suitable for portal hypertension	High toxicity; high mortality; liver zonal specificity; risk of peritonitis	^449^
	TAA-induced	Oxidative stress for centrilobular necrosis, inflammation, thus activating HSCs and inducing fibrosis	6–8 weeks	Simplicity; high reproducibility; similar to alcoholic/cholestatic liver disease	High toxicity; high mortality; long time; significant weight loss	^450^
	DMN-induced	Periportal injury, bile duct proliferation, cholestatic-like fibrosis and inflammation	4–8 weeks	Simplicity; inducing significant fibrosis; short time	Risk of carcinogenesis; high mortality	^451^
	BDL-induced	Bile infarcts, hepatocyte injury, inflammation, cholangiocyte proliferation, HSC activation, and periportal fibrosis	3 weeks	Strong face validity for obstructive diseases; low-cost; short time; ductular reaction	High surgery difficulty; high mortality; nonphysiological pathogenesis	^199^
	Mdr2^-/-^ Mice	Hepatocyte injury, vasodilation, and catheter hyperplasia	Spontaneous animal model (8–12 weeks)	Similar to chronic cholangiopathy; high reproducibility; no external toxin or surgical operation needed	High cost; HCC development; slow progression; background dependency	^452^
	DDC diet	Small bile duct obstruction, biliary epithelial damage, liver injury and ductular reaction	4–8 weeks	Simplicity; excellent reversibility; highly reproducible & nonsurgical	Risk of carcinogenesis; low fibrosis level; heterogeneous fibrosis distribution	^451^
	High-fat diet	Obesity, metabolic syndrome, hepatic steatosis, injury, inflammation, and fibrosis	16 weeks to 12 months	Superior metabolic face validity; comprehensive and progressive pathology	Long time; high cost; variable and mild fibrosis; strain-specific susceptibility	^453^
	CDHFD	Hepatic steatosis, liver injury, and fibrosis	6–24 weeks	High reproducibility; human-relevant NASH fibrosis; noninvasive	Nonphysiological insult; high cost; significant weight loss	^454^
	Alcohol-induced	Steatosis, hepatocyte injury, and inflammation	16–24 weeks	High pathophysiological relevance with alcoholic liver disease	Long time; only mild fibrosis	^455^
	Autoimmune liver fibrosis model	Inflammation	8–12 weeks	Simplicity; low-cost; similar to human immune liver fibrosis; progressive and robust fibrosis	Limited standardization; significant animal morbidity and variability; concomitant pathologies	^456^
Lung	Bleomycin-induced	Inflammation, alveolar structure disruption, and distinct tissue demarcation	4 weeks	Well-established and characterized; simplicity; high reproducibility; low-cost; high human relevance	Severe acute injury; heterogeneous lesion distribution; transient and self-resolving fibrosis	^38^
	Silica-induced	Inflammation, silicotic nodules, progressive massive fibrosis, pleural fibrosis	2–4 weeks	Low-cost; short time; similar to human pneumoconiosis	Low reproducibility; high mortality	^457^
	Asbestos-induced	Inflammation, progressive massive fibrosis, pleural fibrosis	2–4 weeks	Low-cost; short time; high human relevance; unique pathological features	Low reproducibility; high mortality; complex pathology	^458^
	Radiation	Inflammation, collagen deposition, and alveolar septal thickening”	12–24 weeks	Simplicity; similar to human radiotherapy-induced fibrosis	Long time; high mortality; high cost	^73^
	FITC	Inflammation, collagen deposition, and alveolar septal thickening	4 weeks	Real-time monitoring; spatially localized fibrosis	Technical complexity; less characterized; acute inflammatory phase	^459^
	Paraquat	Inflammation, collagen deposition	4 weeks	Simplicity; short time; similar to human IPF; strong oxidative stress insult	High toxicity; high mortality	^460^
	Acid	Increased collagen deposition, vascular permeability, edema, and inflammation	2–4 weeks	Simplicity; high reproducibility; low-cost	High mortality	^461^
	LPS	Acute lung injury, collagen deposition	2–4 weeks	Simplicity; high reproducibility; low-cost; well-characterized mechanism	High mortality; weak and transient fibrosis	^462^
Heart	Angiotensin II	Intramural coronary artery fibrosis, reparative fibrosis, microscopic scarring	2–4 weeks	Simplicity; high reproducibility; short time; high clinical relevance; reliable inducer of cardiac hypertrophy	Nonspecific adverse effects (e.g., CKD); high mortality	^463^
	Isoprenaline	Reparative fibrosis, scarring	2–4 weeks	Simplicity; high reproducibility; short time; high clinical relevance	High toxicity; high mortality; off-target effects	^463^
	MI	Inflammation, collagen deposition	4 weeks	High reproducibility; low cost; inducing significant fibrosis	High surgery difficulty; high mortality; primarily models replacement fibrosis in infarcted regions	^464^
	TAC	Systolic dysfunction, interstitial and perivascular fibrosis	4–8 weeks	Low cost; inducing significant fibrosis; reliable model of hypertrophy	High surgery difficulty; high mortality	^464^
	I/R	Inflammation, collagen deposition	4 weeks	Short time; strong clinical relevance	High surgery difficulty	^464^
	AAC	Blood pressure increased, and collagen deposition	4–8 weeks	High reproducibility; low cost; inducing significant fibrosis	High surgery difficulty; high mortality; may be accompanied by noncardiospecific complications	^465^
	High-salt diet	Inflammation, oxidative stress	4–8 weeks	Simplicity; mimics metabolic abnormalities found in humans	Long time	^466^
Kidney	UUO	Tubular atrophy, interstitial fibrosis	2–4 weeks	Simplicity; high reproducibility; low-cost; short time; recapitulates the typical fibrinogenic response	Low clinical translation rate; high surgery difficulty; impossible to estimate the kidney function from serum	^467^
	I/R	Tubular atrophy, interstitial fibrosis	2–4 weeks	High reproducibility; low-cost; similar to human CKD; the severity of fibrosis can be adjusted according to the duration of ischemia	High mortality; transient and self-resolving fibrosis; male mice tend to be less susceptible to proteinuria and ischemia in this model	^467^
	5/6 nephrectomy	Residual nephron hyperfiltration, glomerulosclerosis, tubulointerstitial fibrosis	12–16 weeks	A good simulation of renal failure after loss of kidney function in humans	High surgery difficulty; long time	^468^
	Adriamycin nephrosis	Focal segmental glomerulosclerosis, tubulointerstitial fibrosis, and inflammation	8–12 weeks	Simplicity; recapitulates human FSGS progression	High toxicity; long time	^469^
	Folic acid	Proximal tubular injury, oxidative stress, and ECM deposition	2–8 weeks	Simplicity; high reproducibility; short time; suitable for studying interstitial fibrosis and AKI to CKD transition	High toxicity	^470^
	Doxorubicin	Glomerular injury, tubulointerstitial fibrosis	4–8 weeks	Simplicity; high reproducibility; short time	Serious side effects such as cardiotoxicity	^471^
	CsA	Tubular injury, interstitial fibrosis	2–4 weeks	Simplicity; short time	High toxicity; high mortality; high cost; a reversible reduction in renal blood flow and glomerular filtration rate	^472^

*TAA* thioacetamide, *ALT* alanine aminotransferase, *AST* aspartate aminotransferase, *DMN* dimethylnitrosamine, *BDL* bile duct ligation, *Mdr2* multidrug resistance protein 2, *DDC* 3,5-diethoxycarbonyl-1,4-dihydrocollidine, *CDHFD* choline-deficient high-fat diet, *HSCs* hematopoietic stem cells, *iPSC* induced pluripotent stem cell, *FITC* fluorescein isothiocyanate, *LPS* lipopolysaccharide, *MI* myocardial infarction, *TAC* transverse aortic constriction, *I/R* ischemia‒reperfusion, *CKD* chronic kidney disease, *AAC* abdominal aortic coarctation, *UUO* unilateral ureteral obstruction, *CsA* ciclosporin A, *AKI* acute kidney injury

**Table 2:** New techniques relevant to fibrosis research

Classification	Level	Application	Technology	Advantage	Examples of discoveries in fibrotic-related diseases
Multiomics sequencing	DNA	Target screening	Whole Genome Sequencing (WGS)	Detect the variations in the coding and noncoding regions of genes	Identified distinct NAFLD molecular subtypes: NAFLD-mSI, -mSII, -mSIII; Linked to hepatic macrophage polarization; Associated with progression risks: cirrhosis and HCC.^473^ Assess genetic-mediated risk of pulmonary fibrosis and predict clinical outcomes.^474^
		Target screening	Whole Exome Sequencing (WES)	Analyze common variations, rare variations, and low-frequency variations; narrow down the research scope and data volume	Rare variations (RVs) related to telomere and surface-active protein genes were discovered, and the genetic risk was explained.^475^
	Epigenetic	Target screening	Whole Genome Bisulfite Sequencing (WGBS)	Gene expression regulation	DNA methylation changes correlated with transcriptional alterations in immune, muscle contraction, and extracellular matrix pathways, identifying specific genes with concordant methylation-expression patterns.^476^
		Target screening	Methylated RNA immunoprecipitation sequencing (MeRIP-seq)	Posttranscriptional modification	Identifying m6A-modified lncRNA E230001N04Rik downregulation in alveolar epithelial cells, correlating with miR-20b-3p/CDK6 dysregulation, senescence, and myofibroblast differentiation in pulmonary fibrosis.^477^
		Target screening	Chromatin immunoprecipitation Sequencing (ChIP-seq)	DNA and protein binding within cells; gene expression regulation; transcription factor binding	Identified GATA4-dependent chromatin accessibility regulation of MYC and PDGFB in liver sinusoidal endothelial cells (LSECs), linking GATA4 loss to increased PDGFB expression and perisinusoidal liver fibrosis.^478^
		Target screening	CUT&Tag Sequencing (CUT&Tag-seq)	DNA and protein binding within cells; gene expression regulation; transcription factor binding	Identified PREP as a nucleus-localized transcriptional coregulator in macrophages, linking PU.1 depletion to exacerbated liver fibrosis progression.^479^
		Target screening	RNA Immunoprecipitation- Sequencing (RIP-seq)	RNA and protein binding within cells; gene expression regulation	RIP-seq identifying LINC00941/lncIAPF binding ELAVL1 to stabilize EZH2/STAT1/FOXK1 mRNAs, inhibiting autophagosome-lysosome fusion and accelerated pulmonary fibrosis progression in IPF.^480^
		Target screening	Assay for transposase-accessible chromatin with high-throughput sequencing (ATAC-seq)	Studying chromatin openness/accessibility; gene expression regulation; transcription factor binding	Elucidated cellular origins and differentiation of human kidney myofibroblasts, identifying distinct subpopulations of pericytes and fibroblasts as key sources of scar-forming myofibroblasts during kidney fibrosis.^248^
		Target screening	Single-cell ATAC sequencing (scATAC-seq)	Studying chromatin openness/accessibility; gene expression regulation; transcription factor binding	scATAC-seq identifying RUNX2 as key transcriptional regulator driving SCUBE2^+^ alveolar fibroblast differentiation into CTHRC1^+^POSTN^+^ pathological fibroblasts in pulmonary fibrosis.^481^
		Target screening	High-throughput chromosome conformation capture (Hi-C)	Chromosome conformation capture; unveiling the 3D structure of the genome	Hi-C technology enabled high-resolution mapping of 3D chromatin interactions (A/B compartments, TADs, chromatin loops) in NAFLD mouse livers, revealing spatial genome reorganization and its association with gene dysregulation.^482^
	RNA	Target screening	Single-cell RNA sequencing (scRNA-seq)	Research on cellular heterogeneity; expression pattern recognition	Revealed specific cell subpopulations (e.g., Foxi1^+^ pulmonary ionocytes, ‘hillocks’, disease-relevant tuft/goblet cell subsets) and their contributions to airway disease states by mapping cellular composition and lineage hierarchy in mouse tracheal epithelium.^483^
		Target screening	Single Nuclei RNA Sequencing (snRNA-seq)	Research on cellular heterogeneity; expression pattern recognition	Revealed a significant expansion of HSC-HSC interactions related to the severity of the disease, which may support the close HSC autocrine interactions and identified NTRK3, an autocrine signaling factor of HSCs, as a potential anti-fibrotic drug target for MASH.^484^
		Target screening	Full-length transcriptome sequencing (Iso-seq)	Isoform exploration; alternative splicing; gene fusion.	Identified lncITPF as an intronic lncRNA upregulated in IPF fibroblasts, interacting with hnRNP-L to affect ITGBL1 expression via histone acetylation.^485^
		Target screening	Long noncoding RNA sequencing (LncRNA-seq)	cell differentiation and development; investigations into regulatory mechanisms;	Identifying m^6^A-modified lncRNA E230001N04Rik downregulation in alveolar epithelial cells, correlating with miR-20b-3p/CDK6 dysregulation, senescence, and myofibroblast differentiation in pulmonary fibrosis.^477^
		Target screening	Circular RNA sequencing (circRNA-seq)	Expression pattern recognition; gene expression regulation; posttranscriptional modification	Identified circDcbld2 as an upregulated candidate circRNA in Kupffer cells during hepatic fibrosis, revealing its role in promoting inflammation, oxidative stress, and HSC activation to drive disease progression.^486^
		Target screening	Small RNA sequencing (smRNA-seq)	Expression pattern recognition; gene expression regulation; posttranscriptional modification	Identified a reproducible murine fecal miRNA profile, and during chronic Trichuris muris infection, revealed differentially expressed miRNAs associated with intestinal fibrosis, wound healing, and barrier integrity.^487^
	Protein	Target screening	Mass spectrometry-based proteomics	Protein‒protein interaction (PPI) network; protein composition and dynamic changes	Identified molecular signatures of fibrosis, inflammation, and steatosis in alcohol-related liver disease, enabling development of diagnostic/prognostic biomarker panels (ROC-AUC 0.92 for significant fibrosis, 0.87 for mild inflammation) that predict liver-related events and mortality.^488^
		Target screening	Antibody-based proteomics	Inflammatory cytokine panel assay; protein biomarker screening	Identifying 140 plasma proteins associated with transplant-free survival in IPF, revealing ECM remodeling/fibroblast activation pathways and enabling machine-learning-derived prognostic signatures.^489^
		Target screening	Metabolomics	Metabolite composition and dynamic changes; protein biomarker screening	Revealing fibrosis-associated dysregulations in amino acid metabolism and lipid metabolism for NAFLD/NASH disease state and progression prediction.^490^
		Target screening	Cytometry by time-of-flight (CyTOF)	Protein biomarker screening; protein identification and quantification; drug discovery	CyTOF analysis revealed 31 distinct immune clusters, including HBV-specific PD-1^+^CD8^+^ tissue-resident memory T cells enriched in tumor borders of HBV^+^ HCC patients, correlating with hepatic damage and fibrosis to characterize disease state and progression.^491^
		Target screening	Cellular indexing of transcriptomes and epitopes by sequencing (CITE-seq)	Expression pattern recognition; protein biomarker screening; protein identification and quantification; cellular heterogeneity	Identified 12 clusters of monocytes and macrophages (resident or recruited) in cardiac immune cells, showing marked changes in abundance between conditions, with resident macrophages found to regulate fibrosis and angiogenesis in early response to cardiac pressure overload.^157^
	Cell	Differentiation and development	Genetic lineage tracing	Expression pattern recognition; cell differentiation and development	Genetic lineage tracing via Scube2-creER identifies alveolar fibroblasts as the dominant origin of injury-induced fibrotic fibroblasts (CTHRC1^+^) and their sequential differentiation trajectory correlating with pulmonary fibrosis progression.^72^
		Differentiation and development	Live fluorescence in situ hybridization (LiveFISH)	Organizational microenvironment; expression pattern recognition; cell differentiation and development	Live FISH techniques visualizing YTHDC1 phase separation facilitating nuclear export of lnc668 and its cytoplasmic translocation correlating with fibroblast-to-myofibroblast differentiation in pulmonary fibrosis.^79^
	Space	Target screening	Spatial transcriptomics	Expression pattern recognition; gene expression regulation; posttranscriptional modification; cell differentiation and development	Uncovered cellular compositions and spatial architectures in COVID-19-induced acute lung injury, revealing dynamic changes linked to inflammation, tissue damage, and repair processes.^492^
		Target screening	Spatial proteomics	Protein biomarker screening; cellular colocalization analysis	Identified fibroblast and macrophage subsets (e.g., SCARA5^+^ myofibroblast progenitors, POSTN^+^/ACTA2^+^ myofibroblasts, IL-1β^+^ proinflammatory macrophages) and their spatial interplay in SSc skin, linking fibrotic niche expansion and clinical severity.^493^
Imaging	Tissue	Target screening	Single-molecule Fluorescence In Situ Hybridization (smFISH)	Protein biomarker screening; cellular colocalization analysis	smFISH confirming foam macrophage aggregation in fibrotic foci with increased cell volume and autofluorescence correlating with radiation-induced pulmonary fibrosis progression.^73^
		Target screening	Fibroblast activation protein inhibitor positron emission tomography (FAPI-PET)	Drug efficacy evaluation; application of precision medicine; molecular diagnosis of diseases	Detected FAP expression in ILD lungs, with SUVtotal correlating with lung function decline, showing scattered distribution in IPF and aggregated in non-IPF, reflecting active fibroblast abundance and aiding in assessing fibrotic activity and predicting lung function changes.^494^
		Target screening	CO-detection by indexing (CODEX)	Protein biomarker screening; cellular colocalization analysis	CODEX-based spatial phenotyping identified CD248^+^ pro-resolving fibroblasts and their neighborhoods with reduced fibrogenic/inflammatory protein expression in IPF, and uncovered functional roles of SERPINE2 (pro-fibrotic) and PI16 (anti-fibrotic) in human lung fibrosis progression and resolution.^74^
Artificial Intelligence (AI)	Tissue	Multimodal data integration	PandaOmics	Target screening; expression pattern recognition; transcription factor binding; cell differentiation and development	Identified TNIK as an anti-fibrotic target by analyzing multiomics data, with generative AI designing TNIK inhibitor INS018_055, which exhibits anti-fibrotic and anti-inflammatory activity in multiple fibrosis models.^446^
		AI-assisted diagnosis system	AI-based measurement of nonalcoholic steatohepatitis (AIM-MASH)	Drug efficacy evaluation; application of precision medicine; research on cellular heterogeneity; molecular diagnosis of diseases	Quantifies MASH histologic features (steatosis, inflammation, ballooning, fibrosis) with high reproducibility, detects treatment-induced fibrosis changes, and predicts disease progression, enabling objective assessment of fibrotic disease states and treatment responses in clinical trials.^495^
		AI-assisted drug design	Generative tensorial reinforcement learning (GENTRL)	Drug synthesis; drug efficacy evaluation	Designed de novo small-molecule DDR1 inhibitors, with two validated in cell-based assays, inhibiting fibrotic markers in lung fibroblasts and collagen expression in hepatic stellate cells.^496^
Model	Tissue microenvironment	Analysis of the organizational microenvironment	3D organoids	Target screening; drug screening; gene expression regulation; cell differentiation and development; cell‒cell interaction	Integrating hepatocytes, cholangiocytes, and mesenchymal cells, recapitulates liver periportal architecture and bile dynamics to simulate cholestatic injury and biliary fibrosis, enabling investigation of cell subtype contributions and disease progression.^497^
		Analysis of the organizational microenvironment	Organ-on-a-chip	Target screening; drug screening; gene expression regulation; cell differentiation and development; cell‒cell interaction	Organ-on-a-chip-based tubuloid cultures model BK virus infection, Wilms tumor, and cystic fibrosis, enabling analysis of cellular heterogeneity and disease-specific features in renal epithelial subpopulations.^498^
		Analysis of the organizational microenvironment	Precision-cut tissue slices	Target screening; drug screening; gene expression regulation; cell differentiation and development; cell‒cell interaction	Precision-cut liver slices from fibrotic rats maintain viable hepatocytes, Kupffer cells, and HSCs/myofibroblasts, enabling in vitro assessment of anti-fibrotic compounds via drug-induced inhibition of α-SMA/pro-collagen 1a1 expression to reflect fibrogenic progression and treatment efficacy.^499^

### Pulmonary fibrosis

#### Epidemiology and etiology

**Fig. 3:**
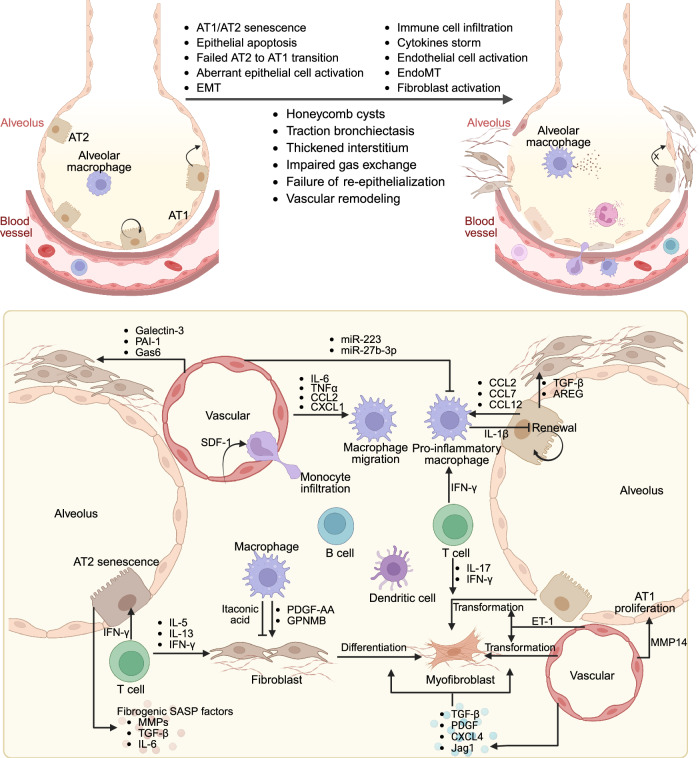
Mechanisms and cellular interactions in pulmonary fibrosis. Following pulmonary injury, AT1 and AT2 cells undergo cellular senescence, apoptosis, aberrant proliferation, EMT, and disrupted AT2-to-AT1 differentiation. These pathological alterations collectively contribute to alveolar structural damage, impaired gas exchange, vascular remodeling, and scar formation. Fibroblasts become activated in response to diverse stimulatory factors. Endothelial cells directly participate in fibrogenesis through EndoMT while concurrently regulating epithelial cell proliferation and transdifferentiation via the paracrine secretion of MMPs, ET-1, TGF-β, and PDGF. Multiple immune cell populations—including T cells, B cells, dendritic cells, and macrophages—infiltrate fibrotic areas. These cells amplify the fibrotic cascade through cytokine release and the modulation of inflammatory responses. EMT, epithelial‒mesenchymal transition; EndoMT, endothelial‒mesenchymal transition; MMPs, matrix metalloproteinases; ET-1, endothelin-1; PDGF, platelet-derived growth factor; SASP factors, senescence-associated secretory phenotype factors; SDF-1, stromal cell-derived factor 1; AT1, alveolar epithelial type I cells; AT2, alveolar epithelial type II cells. This tool was created with BioRender (https://www.biorender.com/)

Pulmonary fibrosis (PF) represents a terminal clinical outcome in numerous respiratory diseases, including IPF, pneumoconiosis, and chronic obstructive pulmonary disease (COPD).^33^ This debilitating condition is characterized by aberrant stromal cell accumulation, excessive ECM deposition, and architectural remodeling of alveolar spaces, collectively driving progressive respiratory failure and high mortality (Fig. 3).^17,34^ As the most prevalent interstitial lung disease, IPF remains etiologically obscure and has a poor prognosis. Its incidence is strongly correlated with advanced age and male predominance, with regional variations: 2.40–2.98 cases in North America, 0.33–2.51 cases in Europe, and 0.57–4.51 cases per 100,000 in Asia-Pacific nations. In England and Wales, IPF has an overall mortality rate of 4.68 per 100,000 person-years, with an annual increase of ~5%.^35^ The median survival for patients ≥65 years is only 2.5–3.5 years post-diagnosis.^36,37^ This high mortality of IPF reflects both therapeutic limitations and diagnostic challenges, including the absence of accessible biomarkers, standardized multidisciplinary diagnostic protocols, and noninvasive monitoring tools for fibrosis progression, highlighting the urgent need for advanced diagnostic modalities. Current pharmacotherapy remains suboptimal; although pirfenidone and nintedanib are FDA-approved for IPF, they merely slow functional decline without reversing established fibrosis.

PF pathogenesis arises from a complex interplay of environmental, genetic, and epigenetic factors. Environmental triggers include viral infections (e.g., SARS-CoV-2), radiation/chemotherapy-induced injury, occupational exposures (e.g., silica, asbestos, coal dust), and airborne environmental toxicants (e.g., smoke, industrial toxins).^38^ Genetic susceptibility also contributes significantly, with familial PF (≥2 relatives with idiopathic interstitial pneumonia) accounting for 5–20% of IPF cases.^39^ The established genetic variants include surfactant dysfunction (*SFTPC*, *SFTPA2*), telomere maintenance defects (*TERT*, *TERC*), and single-nucleotide polymorphisms (*MUC5B*), warranting future genetic profiling for precise disease stratification and targeted interventions.^38^ Furthermore, epigenetic mechanisms, including DNA methylation, histone modifications, and RNA modifications, orchestrate a persistent profibrotic transcriptional program that drives sustained myofibroblast activation and disease progression.^40^

Recently, technological innovations, represented by single-cell sequencing and spatial transcriptomics, as well as multiomics joint analysis, have revolutionized our understanding of PF pathogenesis by delineating the dynamic cellular interplay among the injured epithelium, activated fibroblasts, dysfunctional endothelium, and programmed immune cells.^41^ Critically, pulmonary fibrogenesis represents not only the sum of individual cell behaviors but also highly coordinated multicellular networks and reciprocal signaling cascades. Therefore, systematic dissection of these intercellular communication pathways is essential for both deciphering fundamental disease mechanisms and identifying novel therapeutic targets capable of interrupting these pathological circuits.

#### Role of alveolar epithelial cells in pulmonary fibrosis

The alveolar epithelium consists of two principal cell types: type I (AT1) cells, which facilitate gas exchange and constitute >95% of the epithelial surface area, and type II (AT2) cells, which produce pulmonary surfactant and serve as progenitor cells. Following lung injury, AT2 cells undergo tightly regulated processes of proliferation, transcriptional reprogramming, and differentiation into AT1 cells to restore alveolar architecture and function. Dysregulation of this regenerative program—manifested as aberrant AT2-to-AT1 transition or loss of cellular homeostasis—represents a critical driver of pulmonary fibrogenesis. Single-cell transcriptomic analyses have identified multiple pathological epithelial subpopulations that drive pulmonary fibrosis. A SOX4-governed *KRT5*^−^*KRT17*^+^ epithelial subpopulation originates from dysregulated AT2-AT1 differentiation and exhibits abnormal ECM production.^42^ In murine and human fibrotic lungs, impaired AT2-to-AT1 differentiation leads to the accumulation of pre-alveolar type 1 transitional cells with TP53-mediated senescence and TGFβ-driven DNA damage responses.^43,44^ Most remarkably, airway and alveolar stem cells converge in a persistent *Krt8*^+^ transitional state characterized by p53/NF-kB activation, the expression of cellular senescence markers, and the secretion of profibrogenic factors (e.g., Ctgf, Itgb6, Areg, Hbegf, Edn1, and Lgals3) that potently activate surrounding mesenchymal cells.^45^ These findings are conserved across murine models and human IPF specimens, as confirmed through spatial transcriptomics, lineage tracing, and organoid systems. Molecular drivers include Nkx2-1 deficiency, let-7 miRNA depletion, and epigenetic reprogramming, which lock cells in these pathological intermediate states.^46,47^ Therapeutic strategies targeting cell populations—including phenelzine-mediated AT2 plasticity enhancement, NAD^+^-dependent mitochondrial repair via LNPs, and iPSC-derived airway progenitor transplantation—have demonstrated efficacy in preclinical models by restoring proper differentiation trajectories.^48–50^

In addition to their progenitor functions, AT2 cells actively regulate fibrosis through complex paracrine signaling. Human respiratory airway secretory cells normally guide AT2 regeneration via Notch/Wnt-mediated unidirectional differentiation, but COPD-associated transcriptional dysregulation disrupts this lineage trajectory and promotes aberrant AT2 states linked to fibrotic alveolar remodeling.^51^ AAV6-mediated Wnt/β-catenin activation in AT2 cells enhances regeneration while suppressing fibroblast activation,^52^ whereas UCHL3-dependent p300 deubiquitination drives Ccl2/7/12-mediated M2 macrophage polarization via C/EBPβ.^53^ Mechanical forces also contribute significantly. For example, Cdc42-deficient AT2 cells fail to regenerate alveoli, which leads to elevated parenchymal tension and subsequent activation of TGF-β signaling in stromal cells.^54^ These findings highlight how AT2 cells function as signaling hubs that integrate molecular, cellular, and biophysical cues during fibrogenesis.

Chronic lung injury induces progressive and often irreversible AT2 cell dysfunction through mitochondrial impairment and cellular senescence. Multiple mitochondrial defects contribute to fibrogenesis, including impaired mitophagy regulated by YAP1/TEAD1,^55^ disrupted fatty acid oxidation,^56^ fusion-fission imbalance,^57^ and pathological iron overload.^58^ Pharmacological EP4 receptor activation or mitochondrial transfer from mesenchymal stem cells can partially rescue these deficits.^59^ Parallel investigations of telomere dysfunction have revealed that KLF4-mediated TERT suppression^60^ and FBW7-dependent TPP1 polyubiquitination trigger telomere uncapping and senescence. The therapeutic potential of targeting these pathways is demonstrated by TELODIN peptide inhibition of the FBW7‒TPP1 interaction, which attenuates fibrosis in preclinical models.^61^ Furthermore, environmental exposures, such as PM2.5, upregulate METTL3-mediated m^6^A methylation of STC2 mRNA in airway epithelial cells. This enhances the STC2-mediated inhibition of SQSTM1 ubiquitin-proteasomal degradation, subsequently amplifying mitophagy and aggravating inflammatory fibrotic responses.^62^

EMT represents another important mechanism contributing to fibrotic progression. In both murine and human pulmonary fibrosis, elevated G2 and S phase-expressed protein 1 stabilizes unphosphorylated ZEB1 to drive EMT and promote collagen deposition.^63^ NEK6 kinase mediates the phosphorylation and subsequent degradation of FOXN3, which in turn stabilizes SMAD complexes and enhances fibrogenic transcriptional programs in the lung epithelium.^64^ PRDX1 deficiency creates a vicious cycle of ROS production and TGF-β secretion that simultaneously drives EMT and fibroblast activation.^65^ Additionally, repeated HRV infection induces persistent DNA methylation changes at specific CpG sites in bronchial epithelial cells, fostering a trained immune phenotype that facilitates EMT and extracellular matrix remodeling, thereby accelerating fibrosis.^66^ These findings suggest that targeted inhibition of pathological EMT may represent a viable therapeutic strategy for pulmonary fibrosis.

Alveolar epithelial cells also directly participate in fibrogenesis through complex bidirectional communication with mesenchymal cells. Conditional Nedd4-2 deletion in the murine lung epithelium impairs ENaC-mediated mucociliary clearance while augmenting TGF-β/SMAD signaling, triggering spontaneous pulmonary fibrosis with IPF-like pathology.^67^ Gli1⁺ mesenchymal cells suppress BMP signaling via Hedgehog pathway activation, driving KRT5⁺ basal cell metaplasia, whereas SPARC-rich ECM from IPF fibroblasts limits epithelial migration and promotes fibrosis.^68,69^ Live-cell imaging revealed that IL-6-driven epithelial fluidization via noncanonical SFK/YAP signaling promotes aberrant migration and fibrogenesis, whereas IPF epithelial ERBB-YAP dysregulation delays the transition to activate fibroblasts, which can be rescued by signaling modulation.^70^ In particular, intermediate alveolar stem cells overexpress amphiregulin (AREG), which activates fibroblast EGFR to drive lung fibrosis—a process that can be effectively blocked by AREG-neutralizing antibodies.^71^

These mechanistic insights collectively demonstrate that alveolar epithelial cells serve as central regulators of pulmonary fibrosis through their roles in maintaining progenitor cell competence, coordinating multicellular crosstalk, and responding to microenvironmental stresses. The identification of specific pathological epithelial states and signaling networks provides numerous opportunities for therapeutic intervention. Strategies that preserve epithelial regenerative capacity while preventing maladaptive signaling responses may offer new avenues for clinical translation in this devastating disease.

#### Role of fibroblasts in pulmonary fibrosis

In healthy lung tissue, fibroblasts reside in alveolar walls and septa and function through ECM synthesis and the secretion of critical homeostatic maintenance factors—such as signaling molecules that promote AT2 epithelial cell proliferation and survival.^2^ During pulmonary fibrosis progression and repair, this cell population exhibits significant heterogeneity and plasticity. Single-cell RNA sequencing identified a pathological CTHRC1^+^ fibroblast subpopulation exclusively present in fibrotic lungs (both murine models and human IPF/scleroderma), characterized by increased collagen production, enhanced migratory capacity, and specific localization within fibroblastic foci. Lineage tracing confirmed that these cells originate from alveolar fibroblast differentiation, and remarkably, adoptive transfer experiments demonstrated their ability to drive ECM expansion and disease progression.^72^ Radiation injury models identify another *Hhip*^+^/*Cdh11*^+^/*Pdgfrb*^+^ myofibroblast subpopulation that dominates (>80%) the fibroblast compartment postexposure, orchestrating fibrogenesis by upregulating collagen expression and initiating aberrant collagen-ITGA3 signaling in the capillary endothelium. Concurrently, Col14a1^+^ matrix fibroblasts amplify profibrotic signaling through Angptl/Vegf-mediated crosstalk.^73^ Spatial transcriptomics further revealed two functionally opposed fibroblast subsets: profibrotic Csmd1^+^ cells, which dominate during active fibrogenesis through robust ECM deposition, and antifibrotic Cd248^+^ fibroblasts, which are enriched during resolution phases.^74^ These findings establish fibroblasts as direct pathogenic mediators whose spatiotemporal dynamics critically determine disease progression versus resolution.

The transition to activated myofibroblasts represents a central event in fibrogenesis and is driven by complex molecular reprogramming.^17^ TGF-β1 signaling has emerged as the master regulator of this process, with multiple upstream modulators identified: CRBN and TXNDC5 deficiency attenuate fibrosis by destabilizing TGFBR1 or inhibiting SMAD3 activation,^75,76^ whereas MERTK amplifies profibrotic signaling through an ERK/AKT-SMAD feed-forward loop spanning both fibroblasts and macrophages.^77^ Epigenetic mechanisms further contribute to myofibroblast differentiation. The histone methyltransferase DOT1L promotes pulmonary fibrosis by increasing H3K79 trimethylation at the *Jag1* promoter in fibroblasts, which increases Jagged1 expression and drives profibrotic transformation.^78^ Similarly, YTHDC1-mediated phase separation facilitates the nuclear export of m^6^A-modified lncRNAs, promoting fibroblast activation and exacerbating fibrotic progression.^79^ Notably, the role of transcriptional regulators is context dependent. KLF4 demonstrates cell-specific duality—it promotes myofibroblast transition in PDGFR-β⁺ mesenchymal cells while exerting antifibrotic effects in SMA⁺ cells via CCL2-dependent macrophage recruitment.^80^ Conversely, SERCA2a, tetrameric PKM2, and ACP5 enhance TGF-β signaling through distinct mechanisms, highlighting the intricacy of the pathway.^81,82^ Future research should focus on elucidating the molecular switches governing fibroblast fate decisions and developing strategies to selectively modulate these processes.

#### Role of endothelial cells in pulmonary fibrosis

The pulmonary vascular system is integral to maintaining lung homeostasis, with vascular endothelial cells forming a dynamic interface essential for gas exchange through their close association with alveolar epithelial cells. As pivotal components of alveoli, vascular endothelial cells account for approximately 30% of pulmonary cells in healthy lungs.^83^ A vital characteristic of pulmonary fibrosis is vascular remodeling, accompanied by phenotypic switching and dysfunction of endothelial cells. Both human patients and murine models display a marked reduction in capillary endothelial cells, accompanied by upregulated cell death pathways such as apoptosis and necrosis.^84^ Notably, dying microvascular endothelial cells secrete Gas6 to activate fibroblasts and exacerbate fibrosis.^84^ Further investigations revealed a distinct immune niche in IPF lung tissue composed of immune cells such as T/B cells, dendritic cells (DCs), and PLVAP^+^ bronchial vascular endothelial cells.^85^ Integration of scRNA-seq, spatial transcriptomics, and histopathological analysis demonstrated that diverse lymphocytes within the IPF immune niche are likely recruited through distinct secretory signals from those in PLVAP^+^ bronchial vascular endothelial cells.^85^ Moreover, single-cell transcriptomic network analysis revealed that increased CXCL12 signaling in general capillary endothelial cells represents the most significant alteration in intercellular communication in the lungs of advanced COPD patients, further underscoring the critical role of endothelial cells in regulating inflammation.^86^ Interestingly, the loss of the endothelial transcription factor ERG is strongly associated with aging-related fibrotic changes, including a substantial reduction in pulmonary capillaries.^87^ Similarly, the transcription factor FOXF1 is reduced in the endothelial cells of both human IPF lungs and bleomycin-injured lungs; its deficiency promotes fibroblast proliferation, invasion, and activation while stimulating macrophage migration via the secretion of IL-6, TNFα, CCL2, and CXCL1.^88^ These findings underscore the essential roles of ERG and FOXF1 in maintaining endothelial cell function and promoting tissue repair during lung injury.

Previously recognized as passive conduits for oxygen and nutrient delivery, vascular endothelial cells are increasingly recognized as active regulators of tissue remodeling.^89^ Researchers have revealed that endothelial cells secrete a spectrum of angiocrine factors to orchestrate the vascular microenvironment toward a regenerative or profibrotic phenotype.^90^ In both IPF patients and bleomycin-induced pulmonary fibrosis models, upregulated endothelial MMP19 fosters a profibrotic vascular niche by promoting SDF1-mediated monocyte infiltration and facilitating ET1-induced EndoMT.^91^ Furthermore, endothelial-derived ET-1, TGF-β, PDGF, CXCL4, and JAG1 can also induce fibroblast differentiation, migration, and myofibroblast transformation.^83,92^ Senescent endothelial cells produce galectin-3 to trigger fibroblast-to-myofibroblast transition and NLRP3 inflammasome activation in macrophages.^93^ In addition to secreting profibrotic effectors, endothelial cells also inhibit fibrogenesis by releasing MMP14 to facilitate alveologenesis^94^ and exosomal miRNAs (e.g., miR-223 and miR-27b-3p) to suppress proinflammatory/profibrotic pathways in Flt3^+^ and Tie2^+^ alveolar macrophages (AMs).^95^ Interestingly, a recent study revealed that disorders of the H_2_S-AMPK metabolic axis in endothelial cells can reshape the vascular secretion system and produce the profibrotic vascular secretory factor PAI-1.^96^ These findings suggest the prospect of targeting the H_2_S-AMPK signaling axis to reshape the vascular microenvironment for the treatment of pulmonary fibrosis.

#### Role of immune cells in pulmonary fibrosis

Under steady-state conditions, the lung harbors two distinct populations of tissue-resident macrophages: embryonic-derived AMs and a separate interstitial macrophage population located near large airways and within the lung interstitium. AMs represent the predominant immune cell population in the lung and play a critical role in maintaining pulmonary homeostasis and regulating functional repair.^97^ After injury, recruited monocyte-derived macrophages are abundantly mobilized to the site of tissue damage, where they actively participate in tissue remodeling processes. scRNA-seq of lung tissue from silicotic mice revealed two significantly enriched monocyte subpopulations characterized by high expression of Cxcl10 and Mmp14 and a neutrophil population characterized by high expression of Ccl3. Mechanically, these *Cxcl10*^+^/*Mmp14*^+^ monocytes and *Ccl3*^+^ neutrophils undergo crosstalk through HBEGF-CD44 and CSF1-CSF1R signaling to promote fibrogenesis.^98^ However, the heterogeneity between monocyte-derived macrophages and tissue-resident macrophages and whether transitional states exist between them remain unclear. Recently, a novel SingleR correlation-based hierarchical clustering method identified a unique population of *CX3CR1*^+^*SiglecF*^+^ transitional macrophages with profibrotic properties, which localize within the fibrotic niche and represent a transitional state during the conversion of monocyte-derived macrophages to alveolar macrophages.^99^ Notably, CX3CR1^+^SiglecF^+^ transitional macrophages produce PDGF-AAs to regulate fibroblast proliferation.^99^ In addition, SPP1^+^ macrophages have been observed across multiple models, including human precision-cut lung slices modeling pulmonary fibrosis, SSc-ILD, and IPF.^100,101^ These SPP1^+^ macrophages more closely resembled alveolar macrophages than interstitial macrophages.^100^ SCENIC prediction and chromatin accessibility assays have indicated that transcription factors, including MITF, TFEB, ATF6, SREBF1, BHLHE40, KLF6, ETV5, and/or members of the AP-1 transcription factor family, may regulate profibrotic macrophage differentiation.^102^ These findings suggest the potential of targeting these transcription factors to inhibit the differentiation of SPP1^+^ macrophages to treat multiple forms of pulmonary fibrosis.

Immune cells can produce effector molecules to coordinate with other immune cells in the tissue, as well as with fibroblasts and epithelial cells, to jointly participate in fibrogenesis after injury. For example, macrophages produce itaconic acid to inhibit fibroblast proliferation, whereas GPNMB promotes fibroblast activation.^103,104^ Eliminating B cells can significantly inhibit the differentiation of profibrotic macrophages,^105^ suggesting the profibrotic role of B cells in the pathological process of systemic sclerosis. Furthermore, CD4^+^ T cells release profibrotic cytokines, such as IL-5, IL-13, and IFN-γ, to regulate fibroblast activation.^106,107^ Type I interferon in the microenvironment induces dendritic cell activation and triggers epiregulin release to regulate ECM overproduction.^108^ Emerging mechanisms further highlight the regulatory complexity of immune-mediated fibrosis. PRMT7-mediated histone monomethylation in monocytes upregulates RAP1A expression, promoting monocyte adhesion and migration into lung tissue. This leads to persistent macrophage accumulation and ferroptosis in alveolar epithelial cells, thereby exacerbating fibrotic progression.^109^ These results emphasize the crucial role of the immune microenvironment in pulmonary fibrosis. However, further research is needed to elucidate how other granulocytes (e.g., eosinophils and basophils) and unconventional T cells (e.g., γδ T cells and MAIT cells) contribute to lung tissue remodeling.

### Cardiac fibrosis

#### Epidemiology and etiology

Heart failure (HF) represents a leading cause of mortality worldwide, imposing substantial societal and economic burdens. Epidemiological data indicate that HF affects more than 64 million individuals worldwide, with a prevalence ranging from 1 to 20 cases per 1000 people. This burden is expected to increase due to the aging population and increasing incidence of cardiovascular risk factors.^110^ In 2012, the total economic cost of HF in the United States was estimated at $30.7 billion, with projections suggesting a 127% increase by 2030.^110^ Given these trends, mitigating the socioeconomic impact of HF has become a critical public health priority.

HF is a clinical syndrome characterized by impaired cardiac pumping capacity and circulatory insufficiency, leading to inadequate perfusion of peripheral tissues and failure to meet basal metabolic demands. The etiology of HF is multifactorial and involves diverse pathological mechanisms, such as coronary artery diseases (e.g., myocardial infarction), chronic hypertension, aortic stenosis, arrhythmias, valvular dysfunction, cardiomyopathies, and genetic disorders.^111^ Despite this heterogeneity, cardiac fibrosis emerges as a unifying pathological feature across nearly all progressive forms of HF. The adult mammalian heart exhibits limited regenerative capacity. Following mild or transient injury, resident fibroblasts deposit ECM components to replace necrotic cardiomyocytes, preserving structural integrity and preventing myocardial rupture. However, persistent or severe injury triggers excessive ECM deposition by activated fibroblasts, leading to fibrotic scar formation. This pathological remodeling results in increased myocardial stiffness, reduced compliance, and impaired contractile function.^112^ Additionally, fibrotic tissue disrupts cardiac electrophysiology by slowing action potential propagation, thereby predisposing patients to fatal arrhythmias and conduction abnormalities.^113^

**Fig. 4:**
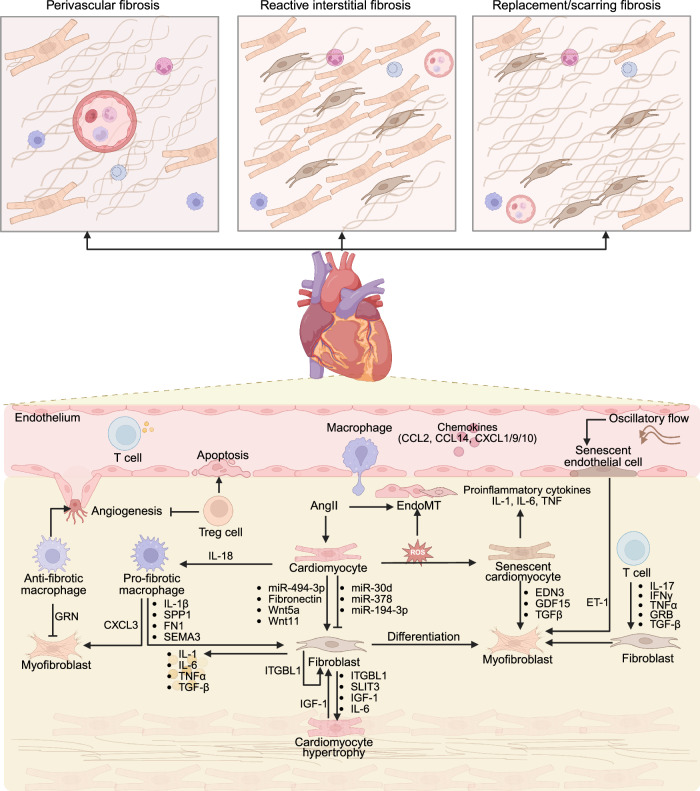
Mechanisms and cellular interactions in cardiac fibrosis. Myocardial fibrosis can be classified into three distinct types: infiltrative perivascular fibrosis, reactive interstitial fibrosis (where cardiomyocytes are preserved without significant loss), and replacement fibrosis (characterized by substantial cardiomyocyte loss followed by substitution with fibroblasts and the extracellular matrix). Mechanistically, damaged cardiomyocytes release various profibrotic factors, promoting the activation of fibroblasts and inducing proinflammatory macrophages. Injured cardiomyocytes increase ROS levels, which in turn induce EndoMT. In addition, cardiomyocytes can release antifibrotic exosomal miRNAs to inhibit the proliferation and activation of fibroblasts. Conversely, fibroblasts regulate cardiomyocyte hypertrophy and induce chronic inflammatory responses by releasing profibrotic effector molecules. Senescent endothelial cells and cardiomyocytes release SASP factors to regulate myofibroblast activation and ECM deposition. The chemokines present in the environment recruit many T cells, macrophages, etc., and participate in regulating the activation of myofibroblasts and fibroblasts, endothelial apoptosis, and angiogenesis. EndoMT, endothelial-to-mesenchymal transformation; IGF-1, insulin-like growth factor 1; GRN, granulin; SPP1, secreted phosphoprotein 1; FN1, fibronectin 1; SEMA3, semaphorin 3; ITGBL1, integrin beta-like protein 1; SLIT3, slit guidance ligand 3; EDN3, endothelin 3; ET-1, endothelin-1; GDF15, growth differentiation factor 15; GRB, Granzyme B. This tool was created with BioRender (https://www.biorender.com/)

On the basis of etiology and clinical manifestations, cardiac fibrosis can be classified into three distinct subtypes: reactive interstitial fibrosis, infiltrative perivascular fibrosis, and replacement fibrosis (Fig. 4). Reactive interstitial fibrosis is characterized by diffuse ECM deposition within the myocardial interstitium without cardiomyocyte loss and is typically driven by chronic pathological stimuli (e.g., inflammation and aging).^114^ Infiltrative perivascular fibrosis manifests as collagen accumulation predominantly around the coronary microvasculature and is common in hypertensive heart disease.^114^ Replacement fibrosis occurs as a reparative response to cardiomyocyte necrosis, often following acute ischemic injury.^114^

While fibroblasts and myofibroblasts serve as the primary offenders of fibrotic scar formation, multiple cardiac cell populations, including cardiomyocytes, endothelial cells, and immune cells, undergo phenotypic and functional reprogramming under pathological stress.^115^ These cells synergistically modulate fibrotic progression through two key mechanisms: (1) the secretion of paracrine effector molecules and (2) the activation of mesenchymal transition pathways. Deciphering the spatiotemporal heterogeneity of cellular phenotypes and functional states during fibrotic remodeling is essential for elucidating the molecular underpinnings of cardiac fibrosis and identifying novel therapeutic targets.

#### Role of cardiomyocytes in cardiac fibrosis

Cardiomyocytes, the most abundant and important cells in cardiac tissue, are responsible for regulating contraction, electrical conduction, and mechanical stress adaptation. Cardiomyocytes exhibit significant regional and functional heterogeneity, as evidenced by distinct gene expression profiles between the atrial and ventricular subtypes.^116^ During pathological stress, such as heart failure, cardiomyocytes undergo transcriptional reprogramming characterized by the downregulation of *MYH6* and the upregulation of stress-responsive genes (e.g., *NPPA*, *NPPB*, *MYH7*, and *ACTA1*).^116^ However, these changes vary across different cardiomyopathies. For example, pressure overload-induced hypertrophy triggers the upregulation of ERBB4 and TBX20 expression, which induces CM proliferation and regeneration.^117^ Interestingly, hypertrophic cardiomyocytes exhibit an imbalanced angiogenic profile, marked by elevated proangiogenic VEGFA but suppressed mature VEGFB, potentially leading to dysfunctional neovascularization.^117^ Furthermore, hypertrophic cardiomyopathy is associated with widespread upregulation of ECM-related genes (*LUM*, *DCN*, *FN1*, *CTGF*, and *COLIA2*) across all subpopulations of cardiomyocytes, as well as the ECM organization pathway.^118^ A spatial multiomics atlas of human myocardial infarction revealed that stressed ventricular cardiomyocytes highly express ANKRD1 and NPPB, which are located primarily below the transition zone within the injured area.^119^

In cardiomyocytes, a variety of enzymes that modulate epigenetic and posttranslational modifications participate in regulating cell functions. Among these deacetylases, the sirtuin family, NAD^+^-dependent deacetylases, has attracted significant attention. Studies have demonstrated that SIRT7-mediated deacetylation of the transcription factor GATA4 represses the transcription of prohypertrophic and fibrotic genes.^120^ On the other hand, SIRT2 activates the AMPK signaling pathway through the deacetylation of LKB1, ameliorating both aging- and angiotensin II-induced myocardial hypertrophy.^121^ Downregulation of SIRT6 in heart failure patients results in increased H3K9ac acetylation, leading to aberrant activation of the IGF signaling pathway.^122^ In contrast to the protective role of SIRT2/3/6, SIRT4 promotes myocardial hypertrophy by inhibiting the interaction between MnSOD and SIRT3. This leads to increased acetylation of MnSOD, resulting in ROS accumulation.^123^ These findings underscore the therapeutic potential of targeting sirtuin-mediated protein modifications and epigenetic alterations to ameliorate myocardial fibrosis.

Cardiomyocyte‒fibroblast crosstalk has been demonstrated to be pivotal in pathological cardiac remodeling, and extracellular vesicles loaded with microRNAs (miRNAs) released by cardiomyocytes are important messengers. For example, cardiomyocyte-derived exosomal miR-30d suppresses fibroblast proliferation and activation and improves cardiac function in ischemic models. Low expression of miR-30d in the extracellular vesicles of the heart and plasma is associated with poor remodeling in patients with heart failure.^124^ miR-194-3p, which is decreased in diabetic cardiomyopathy, inhibits TGF-β-mediated fibroblast-to-myofibroblast conversion.^125^ Conversely, cardiomyocyte-released miR-494-3p-enriched exosomes contribute to pressure overload-induced cardiac fibrosis by promoting fibroblast activation, with its transcription downregulated by cardiomyocyte-specific deletion of Peli1.^126^ These findings position cardiomyocyte-derived miRNAs as potential biomarkers and therapeutic targets for cardiac fibrosis.

In addition to miRNAs, cardiomyocytes influence fibrosis through direct protein secretion and signaling cascades. Cardiomyocyte-secreted fibronectin regulates fibroblast proliferation and activation in a FAK signaling-dependent manner.^127^ Inhibition of fibronectin transcription by SENP1 overexpression or HSP90ab1 Lys72 mutation attenuates postinfarction fibrosis.^127^ Pressure overload reduces low-density lipoprotein receptor-related protein 6 expression in cardiomyocytes, destabilizing β-catenin and increasing Wnt5a/Wnt11 release, which in turn increases the expression of TGF-β, collagen I, and collagen II in fibroblasts.^128^ Senescent cardiomyocytes secrete senescence-associated secretory phenotype (SASP) factors that modulate local inflammatory responses (e.g., IL-1, IL-6, and TNF) and promote myofibroblast activation (e.g., EDN3, GDF15, and TGF-β).^129^ Moreover, NOX4 upregulation of mitochondrial ROS in cardiomyocytes induces profibrotic embryonic-derived resident CCR2^−^MHCII^hi^CX3CR1^hi^ macrophage activation through the release of IL-18.^130^

#### Role of fibroblasts in cardiac fibrosis

In adult cardiac tissue, fibroblasts account for 15–24% of all cardiac cells and are crucial in regulating cardiac remodeling by generating the ECM.^131^ Elucidating the dynamic activation of fibroblasts at the single-cell level and screening key regulatory molecules are conducive to improving fibrosis by blocking excessive ECM deposition. Recent single-nucleus profiling revealed key genes, including *POSTN*, *AEBP1*, *COL22A1*, *JAZF1*, and *PRELP*, that regulate fibroblast activation.^132^ In patients with hypertrophy and heart failure, snRNA-seq identified a *POSTN*^+^*RUNX1*^+^*CILP*^+^*AEBP1*^+^ fibroblast subpopulation,^133^ whereas an angiotensin II-induced heart failure model revealed a fibrogenic *Cilp*^+^ fibroblast subset^134^, highlighting the potential of targeting *CLIP*^+^ fibroblasts. Further insights from integrated snRNA-seq and snATAC-seq analyses in myocardial infarction patients revealed a pseudotemporal trajectory from *SCARA5*^+^ fibroblasts (Fib1) to profibrotic *POSTN*^+^*COL1A1*^+^*FN1*^+^ fibroblasts (Fib2).^119^ Network analysis highlighted KLF4 as a key transcriptional suppressor of Fib1 activation, whereas TEAD3, GLI2, and RUNX2 were identified as critical regulators of Fib2-mediated fibrotic progression.^119^ These findings suggest that KLF4 may inhibit fibroblast activation, while targeting TEAD3, GLI2, and RUNX2 could ameliorate fibrosis progression. Interestingly, single-cell sequencing revealed an antifibrotic myofibroblast subset at 7 days post-myocardial infarction characterized by high expression of *Htra1* and *Htra3*, which inhibit the TGF-β signaling pathway, as well as the *Wisp2* and *Sfrp1* genes, which suppress fibroblast proliferation and fibrosis.^135^ This underscores the remarkable heterogeneity of fibroblast populations, not only between heart failure patients with distinct etiologies but also within individual patients. Integrated multiomics analysis across diverse heart failure populations may facilitate the identification of profibrotic fibroblast biomarkers or regulatory factors that drive myocardial fibrosis.

Fibroblasts also influence cardiac remodeling through secretory signaling. Integrin beta-like 1 (ITGBL1) is highly expressed in fibroblasts across various heart diseases.^136^ Fibroblast-derived ITGBL1 not only autocrinely enhances TGF-β signaling to activate fibroblasts but also paracrinely regulates Wnt signaling in cardiomyocytes, thereby inducing cardiac hypertrophy.^136^ Fibroblast and vascular mural cell-released SLIT3 induces cardiomyocyte hypertrophy and upregulates hypertrophy-related genes in pressure overload-induced cardiac remodeling.^137^ Senescent fibroblasts upregulate proinflammatory (IL-1, IL-6), profibrotic (TNF-α, TGF-β) cytokines, and IGF-1, which modulates cardiomyocyte hypertrophy.^129^ In response to injury, fibroblast-specific p38α MAP kinase promotes cardiac hypertrophy by secreting the paracrine factor IL-6.^138^ Therefore, these findings indicate that in addition to their pivotal role in fibroblast proliferation, activation, and differentiation in fibrosis, their interactions with other cardiac cells are equally critical for cardiac remodeling.

#### Role of endothelial cells in cardiac fibrosis

As the most abundant cell type among cardiac interstitial cells, ECs account for approximately 24% of the cells involved in homeostasis. Cardiac ECs can be divided into endocardial ECs, vascular ECs, and lymphatic ECs.^131^ Vascular ECs demonstrate remarkable transcriptional heterogeneity along the arterial‒capillary‒venous axis, with single-cell analyses revealing five distinct subpopulations: arterial, arterial‒capillary, capillary, venous‒capillary, and venous ECs.^139^ This spatial specialization enables precise regulation of vascular function but also creates vulnerability to pathological remodeling. In ischemic injury, ECs undergo significant phenotypic alterations characterized by the upregulation of plasmalemma vesicle-associated protein (PLVAP) in CD31^+^ cells bordering infarct zones. While PLVAP modulation represents a potential therapeutic target for enhancing neovascularization, its pleiotropic effects on vascular permeability and leukocyte trafficking necessitate cautious therapeutic development.^140^ Recent multiomics approaches have revealed a profibrotic EC subset characterized by ACKR1 and CCL14 overexpression within fibrotic niches, which orchestrates leucocyte extravasation and subsequent ECM deposition.^141^ The conserved role of ACKR1^+^ ECs across organ systems, as demonstrated in skin fibrosis models, suggests that targeting this subpopulation may offer a promising strategy for mitigating cardiac fibrosis while minimizing off-target effects.^142^

Endothelial dysfunction has emerged as a central driver during cardiac fibrotic remodeling through multiple interconnected mechanisms. Mechanotransduction pathways, which are mediated by TRPV4, Kir2.1, and PIEZO1 sensors, become dysregulated in hypertension and heart failure, impairing the ability of ECs to adapt to hemodynamic stresses.^143^ Genetic variants in these mechanosensors, particularly PIEZO1 mutations, may predispose patients to cardiovascular pathologies by disrupting force-dependent signaling.^144^ Senescent ECs contribute to cardiac fibrosis by producing the SASP factor endothelin-1 to trigger collagen synthesis in cardiac fibroblasts.^129^

EndoMT, a process in which ECs lose their endothelial identity and acquire mesenchymal features, is closely related to heart failure and fibrotic progression. Disruption of autophagy, accumulation of ROS, and H_2_S deficiency have all been implicated in EndoMT-mediated fibrosis.^145,146^ Therapeutic strategies targeting these pathways, such as endothelial-specific overexpression of cystathionine γ-lyase (H_2_S-producing enzyme) and modulation of the AGE/RAGE-autophagy axis, have shown promise in decreasing EndoMT in vivo.^145,147^ Intriguingly, emerging evidence indicates that EndoMT regulation extends beyond canonical environmental factors (e.g., TGF-β and IL-6) to encompass metabolic modulation.^148^ For example, lactic acid accumulation in the postinfarction microenvironment promotes EndoMT via Snail1 lactylation.^149^ SIRT1 has shown great potential in regulating EndoMT to improve myocardial fibrosis, including by inhibiting the TGF-β/SMAD2/3 pathway and mediating the deacetylation and degradation of NICD.^120,150^

#### Role of immune cells in cardiac fibrosis

In adult cardiac homeostasis, immune cells constitute approximately 5% of the myocardial cellular population, with macrophages being the predominant cell type.^116^ Additional immune cell populations include T cells, B cells, mast cells, DCs, and others. Macrophages account for nearly 50% of cardiac immune cells and play crucial roles in maintaining cardiac homeostasis by participating in inflammation, phagocytosis, electrical conduction, and tissue repair.^151^ During cardiac remodeling, macrophages exhibit significant temporal and spatial heterogeneity, leading to dynamic shifts in their subsets and functions. scRNA-seq analyses of human dilated cardiomyopathy (DCM) and ischemic cardiomyopathy (ICM) hearts revealed a tissue-resident *CXCL8*^h*i*^*CCR2*^+^*HLA*^*-*^*DR*^hi^ macrophage subset enriched in severely fibrotic areas, where it interacts with immunoregulatory *ACKR1*^+^ ECs.^152^ Platelet-derived CXCL4 drives profibrotic *SPP1*^+^ macrophage differentiation in vivo. Ligand‒receptor interaction analyses further revealed that *SPP1*^+^ macrophages orchestrate fibroblast activation by secreting SPP1, FN1, and SEMA3.^153^ Additionally, infiltrated CX3CR1^+^ macrophages secrete IL-1β to activate myofibroblasts in a BRD4-dependent manner.^154^ Conversely, Bhlhe41^+^ macrophages specifically localize to developing infarct areas in myocardial infarction models, where they help limit infarct expansion by secreting Grn to counteract the effect of Tnf-α on Tnfrsf1a, thereby inhibiting myofibroblast activation.^155^ Interestingly, cardiac-resident macrophages can also exert antifibrotic effects by stimulating angiogenesis and reparative immune responses.^156,157^ Therefore, indiscriminate macrophage depletion as a therapeutic strategy for fibrosis carries certain risks, and targeting functionally specialized regulatory molecules may be a better option. For example, YAP/TAZ functions as an important regulator of the macrophage phenotype transition between proinflammatory and reparative states. Genetic deletion of YAP/TAZ alleviates cardiac fibrosis by enhancing the reparative response in macrophages.^158^ Moreover, improving macrophage efferocytosis and reshaping macrophage metabolism to foster a prorepair immune microenvironment also provides opportunities for myocardial fibrosis therapy.^159^

Following cardiac injury, chemotactic signals such as NLRP3 inflammasome activation, CCL2, GM-CSF, IL-6, and CXCL1/9/10 recruit T cells to the injured myocardium.^160^ Infiltrating T cells contribute to fibrosis by releasing profibrotic cytokines, including IL-17, IFN-γ, and TNF-α.^160^ In addition, CD8^+^ cytotoxic T lymphocyte-secreted degranulated granzyme B directly contributes to fibroblast-to-myofibroblast transformation.^161^ Regulatory T cells (Tregs) exhibit dual profibrotic effects: they induce capillary rarefaction via antiangiogenic effects and endothelial apoptosis while secreting TGF-β to stimulate ECM accumulation.^162^ Notably, MyD88 has emerged as a key modulator of T-cell activation, with studies demonstrating its role in reducing cardiac inflammation, suggesting the potential of MyD88 in antifibrosis treatment.^163^

### Liver fibrosis

#### Epidemiology and etiology

Liver fibrosis represents a critical pathological consequence of persistent hepatic inflammation and injury, serving as a common precursor to cirrhosis and hepatocellular carcinoma (HCC), two leading causes of global morbidity and mortality. Epidemiological studies estimate that 7.3% of the general population exhibits significant liver fibrosis, with 3.3% progressing to advanced fibrosis and 1.3% developing cirrhosis.^164^ The increasing incidence of chronic liver disease has led to approximately 58.41 million new cases and 1.4 million deaths annually worldwide, underscoring liver fibrosis as a major public health challenge.^165^ Despite its clinical importance, therapeutic options remain severely limited. To date, only Resmetirom and Semaglutide have been recently approved for the treatment of adults with noncirrhotic MASH with moderate to advanced liver fibrosis.^166^ This stark lack of effective treatments highlights the urgent need to elucidate the pathophysiology and underlying mechanisms driving liver fibrosis progression.

The primary causes of liver fibrosis vary geographically and socioeconomically. Chronic viral infections (hepatitis B and C) remain the most common etiological factors, inducing acute or chronic hepatitis with subsequent hepatocyte death.^165^ Additionally, alcohol consumption, exposure to hepatotoxic chemicals, hepatic congestion, parasitic infections, and chronic cholangiopathies contribute significantly to liver fibrogenesis.^167^ In recent decades, the global obesity epidemic has accelerated the prevalence of MASLD, which has become the leading cause of liver fibrosis in developed countries.

**Fig. 5:**
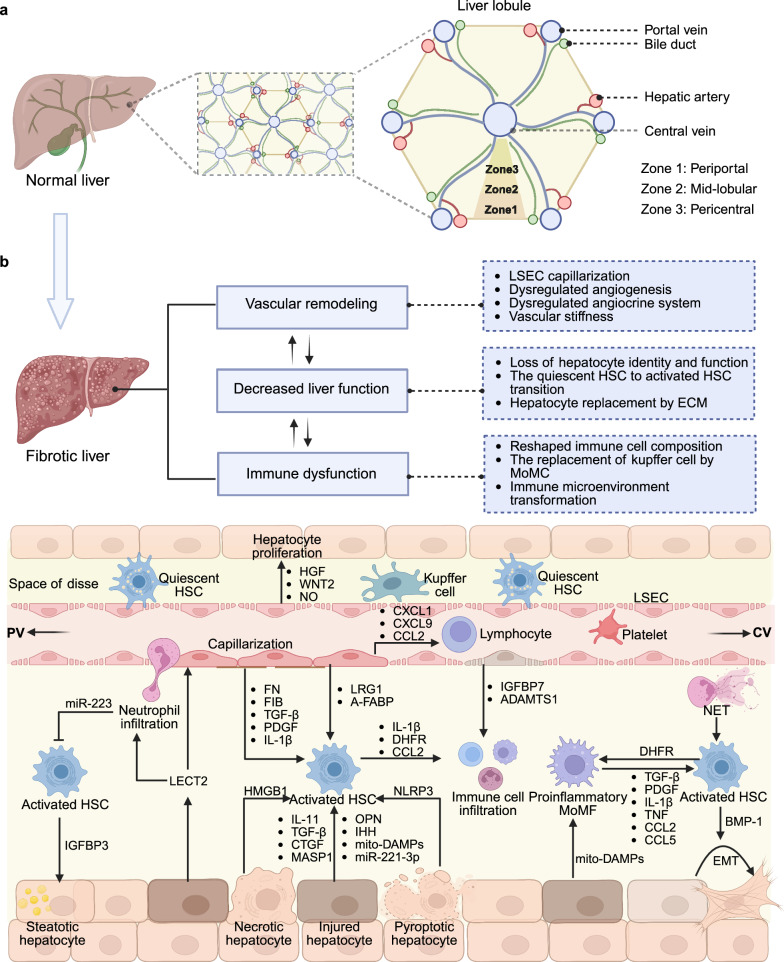
Mechanisms and cellular interactions in liver fibrosis. **a** Geometric representation of a liver lobule. The structure consists of cords of hepatocytes typically organized in a hexagonal shape around the central vein. The vertices of the liver lobule represent the portal triad area, which contains branches of the hepatic artery, bile duct, and portal vein. Hepatocytes within the lobule exhibit metabolic zonation and are divided into three distinct zones (periportal, midlobular, and pericentral). **b** Pathological progression and cellular interactions of liver fibrosis. Liver fibrosis mainly involves vascular remodeling, decreased liver function, and immune dysfunction. Vascular remodeling is induced by LSEC capillarization, which further induces dysregulated angiogenesis, a dysregulated angiocrine system, and increased vascular stiffness. Decreased liver function is caused mainly by the loss of hepatocyte identity and function, the activation of HSCs, and ECM overaccumulation. The reshaped immune cell composition, replacement of Kupffer cells by MoMC, and immune microenvironment transformation commonly promote immune dysfunction. During liver fibrosis, liver cells engage in crosstalk with each other to regulate liver fibrosis progression. Injured hepatocytes initially secrete LECT2 to promote neutrophil infiltration and LSEC capillarization. Injured hepatocytes also promote HSC activation by releasing a series of profibrotic and proinflammatory cytokines (e.g., IL-11, TGF-β, CTGF, and HMGB1) and regulating proinflammatory MoMFs via the production of mito-DAMPs. Moreover, injured hepatocytes can transform into fibroblasts via EMT promoted by BMP-1 from activated HSCs. During fibrosis, LSECs undergo capillarization and promote HSC activation via paracrine factors (e.g., FN, FIB, TGF-β, PDGF, IL-1β, LRG1, and A-FABP), recruit immune cells via chemokines (CXCL1, CXCL9, CCL2, IGFBP7, and ADAMTS1), and accelerate hepatocyte proliferation via HGF, WNT2, and NO. The recruited immune cells subsequently release factors (e.g., TGF-β, PDGF, CCL2, and CCL5) to accelerate HSC activation and secrete miR-223 to inhibit HSC activation, which commonly regulates HSC activation. Activated HSCs, as the main producers of ECM, influence immune cell infiltration via IL-1β, DHFR, and CCL2. LSEC liver sinusoidal endothelial cell, HSC hepatic stellate cell, ECM extracellular matrix, MoMC monocyte-derived multipotential cell, LECT2 leukocyte cell-derived chemotaxin 2, CTGF connective tissue growth factor, HMGB1 high mobility group box-1, NLRP3 Nod-like receptor protein 3, MASP1 mannan-binding lectin serine protease 1, OPN osteopontin, IHH Indian hedgehog, mito-DAMPs mitochondria-derived damage-associated molecular patterns, EMT epithelial‒mesenchymal transition, BMP-1 bone morphogenetic protein-1, FN fibronectin, PDGF platelet-derived growth factor, LRG1 leucine-rich alpha-2-glycoprotein 1, A-FABP adipocyte fatty acid binding protein, CXCL C-X-C motif ligand, CCL C-C motif ligand, IGFBP7 insulin-like growth factor-binding protein 7, ADAMTS1 a disintegrin and metalloproteinase with thrombospondin motifs, TNF tumor necrosis factor, DHFR dihydrofolate reductase. This tool was created with BioRender (https://www.biorender.com/)

Liver fibrosis refers to a dynamic and complex pathological process characterized by a series of cellular and microenvironmental events. The progression begins with hepatocyte injury, which is subsequently accompanied by the recruitment and infiltration of immune cells, liver sinusoidal endothelial cell (LSEC) capillarization, and hepatic stellate cell (HSC) activation, which together lead to ECM deposition, destruction of the liver architecture, and induction of portal hypertension.^168^ While HSC activation is the central driver of liver fibrosis, emerging evidence highlights the critical role of intercellular crosstalk, wherein other liver cell types either promote or suppress HSC activation. The liver is structurally organized into functional lobules, with the hexagonal architectural units centered around the central vein. At the periphery of the lobule lies the portal triad, which is composed of branches of the hepatic artery, portal vein, and bile ducts.^169^ Blood, which is rich in oxygen from the hepatic artery and rich in nutrients from the portal vein, is filtered through the sinusoids toward the central vein. Hepatocytes within the lobule exhibit metabolic zonation, which is traditionally divided into three distinct zones (periportal, midlobular, and pericentral), each with specialized functions (Fig. 5a).^170^ Recent studies have revealed that zonation is not exclusive to hepatocytes but extends to HSCs, LSECs, and immune cells, suggesting a spatially coordinated regulatory network.^171^ However, how zonation dynamics shift during liver fibrosis progression remains poorly understood. Advances in single-cell genomics, including single-cell and nuclear RNA sequencing and the rapidly evolving fields of spatial transcriptomics and proteomics, are now providing unprecedented insights into cellular heterogeneity and zonation alterations in fibrotic liver disease, offering novel strategies for liver fibrosis treatment.

#### Role of hepatocytes in liver fibrosis

Hepatocytes, the primary parenchymal cells of the liver, constitute approximately 60% of the liver’s cellular composition and serve as the functional units responsible for energy metabolism, detoxification, and protein synthesis.^168^ Positioned between liver capillaries and sinusoids within the lobular architecture, hepatocytes exhibit remarkable regenerative capacity, enabling rapid tissue repair following injury. However, in chronic or severe damage, hepatocytes shift from a regenerative role to a profibrotic role, actively contributing to liver fibrosis through multiple mechanisms. The spatial organization of hepatocytes profoundly influences their fibrogenic potential. Traditional metabolic zonation divides hepatocytes into three functionally distinct populations: Periportal (Zone 1), which expresses *Pck1*, *Hal*, *Gls*, *Hsd17b13*, *Ass1*, and *Arg1* and specializes in glucogenesis, protein secretion, ureagenesis, and β-oxidation;^172^ Midlobular (Zone 2), which expresses *Hamp* and *Igfbp2* without clear metabolic specialization; and Pericentral (Zone 3), which expresses *Cyp3a11*, *Adh4*, *Glul*, and *Bche* and is responsible for fatty acid synthesis, glycolysis, lipogenesis, and ketogenesis. Recent spatial transcriptomics has revealed dynamic shifts in hepatocyte zonation during fibrosis, with the emergence of two novel hepatocyte clusters (marked by *Pnpla3*, *Cpeb4*, and *Hsp90aa1*) that disrupt classical zonation patterns.^173^ The transcription factor Tcf7l2 has been identified as a critical regulator of pericentral identity (Zone 3), with its dysfunction leading to defective bile acid synthesis, dysregulated glutamine homeostasis, and exacerbated diet-induced liver fibrosis.^174^

In response to injury, hepatocytes initiate fibrogenesis through multiple parallel pathways.^9^ Injured hepatocytes release a complex array of profibrotic mediators, including canonical cytokines (e.g., TGF-β and CTGF), novel fibrogenic effectors (IL-11),^175^ miRNA-enriched exosomes (miRNA-221-3p), iron-loaded extracellular vesicles (EVs) that induce oxidative stress,^176^ and MASP1-containing small EVs that directly activate HSCs (Fig. 5b).^177^ While controversial, some evidence indicates that hepatocytes can undergo partial EMT under chronic injury, adopting a myofibroblast-like phenotype capable of producing ECM and contributing to liver regeneration.^178^ When dysregulated, however, this process shifts toward excessive fibrogenesis, accelerating liver fibrosis. More definitively, dying hepatocytes release multiple damage-associated molecular patterns (DAMPs), namely, mitochondria-derived DAMPs (mito-DAMPs), which trigger inflammation and HSC activation;^179^ HMGB1, which modulates HSC autophagy;^180^ and NLRP3 inflammasome components, which trigger IL-1β secretion and induce fibrosis.^181^ Stressed hepatocytes upregulate E4BP4, driving osteopontin secretion via YAP signaling to activate HSCs.^182^

In addition to directly activating HSCs, hepatocytes can promote liver fibrosis by modulating immune responses and LSEC function. For example, hepatocyte-released CCL9 creates a profibrotic microenvironment that recruits macrophages, promotes M1 polarization, and regulates inflammatory cytokine responses.^183^ Notably, leukocyte cell-derived chemotaxin 2 (LECT2) secreted from injured hepatocytes plays dual roles as both a neutrophil chemoattractant and a direct activator of LSEC dysfunction. LECT2 binds specifically to the orphan receptor Tie1 on LSECs, activating PPAR signaling and promoting LSEC capillarization.^184^ Moreover, LECT2 was originally identified as a chemotactic factor for neutrophils and induces an inflammatory response.^185^ The pleiotropic effects of mediators such as LECT2, which act simultaneously on immune cells and endothelial cells and subsequently affect HSCs, highlight the complexity of cell‒cell communication in liver fibrosis and suggest that therapeutic strategies may need to target multiple pathways simultaneously for optimal efficacy.

#### Role of hepatic stellate cells in liver fibrosis

HSCs are the primary source of matrix protein-secreting myofibroblasts, which play a central role in liver fibrosis through a process commonly termed “trans-differentiation” or activation. Accounting for 5–10% of resident liver cells, HSCs reside in the subendothelial space of Disse, interposed between LSECs and hepatocytes.^21^ In a healthy liver, HSCs maintain a quiescent, nonproliferative phenotype and store 50–80% of the total vitamin A in the body.^186^ However, upon persistent liver injury, HSCs undergo activation, transforming from vitamin A-storing cells into proliferative, contractile, and chemotactic myofibroblasts. Recent studies in single-cell technologies have highlighted the remarkable heterogeneity and spatial zonation of HSCs in both healthy and fibrotic livers. HSCs in normal liver tissue can be classified into two major clusters: CD74^+^PDGFRB^+^ HSCs, which are enriched in periportal regions and contribute to biliary and vascular stability, and CD74^-^PDGFRB^+^ HSCs, which are scattered throughout the lobules and maintain liver architecture.^173^ Further refinement via single-cell RNA sequencing has identified spatially distinct subpopulations, including portal vein-associated HSCs (PaHSCs, marked by *Ngfr*) and central vein-associated HSCs (CaHSCs, marked by *Adamtsl2*).^187^ Notably, CaHSCs, which specifically express Lpar1, have been identified as the dominant collagen-producing cells in centrilobular fibrosis. Another study revealed a specialized Zone 1-HSC subpopulation that migrates from periportal regions to central veins during fibrosis, contributing to sinusoidal capillarization without expressing typical myofibroblast markers such as α-SMA or collagen 1.^186^ Additional classifications include GPC3^+^HSCs, concentrated portal and central veins involved in glycosaminoglycan metabolism, and DBH^+^ HSCs, which are located perisinusoidally and function as antigen-presenting cells.^188^ Lineage-tracing studies have demonstrated that a small subset (~10%) of HSCs marked by transcription factor 21 (*Tcf21*) becomes activated upon injury and generates 62%-67% of all myofibroblasts in fibrotic livers.^189^ Furthermore, MFAP4^+^ HSC clusters exhibit functional diversity in liver fibrosis.^190^ These findings suggest that targeting specific HSC subpopulations could lead to more effective antifibrotic therapies.

In addition to being activated by profibrotic and proinflammatory cytokines, emerging evidence highlights the importance of metabolic reprogramming, epigenetic alterations, and posttranslational modifications in HSC activation. To meet the heightened metabolic demands of myofibroblasts, activated HSCs shift toward glutaminolysis and aerobic glycolysis driven by Hedgehog signaling.^191^ Increased glucose transporter 1, glucose uptake, and glycolysis can directly promote HSC activation.^192^ Epigenetic regulators such as histone and DNA methyltransferases such as G9a and DNMT1 facilitate gluconeogenic gene expression and mitochondrial function, reinforcing fibrogenic activation.^193^ DNA methylation-mediated suppression of PPARγ facilitates STAT3-driven metabolic reprogramming and lipid depletion during early HSC activation, whereas RNA m^6^A modifications of major collagen genes contribute to massive ECM production in later stages.^194^ Increased glycolysis in HSCs also amplifies fibrosis by promoting fibrogenic EV release in the pericentral zone, which is mediated by the upregulation of EV-related genes such as RAB31 through H3K9 acetylation.^195^ Posttranslational modifications of key fibrotic regulators also play critical roles. SIRT6 attenuates liver fibrosis by deacetylating SMAD2, thereby suppressing TGF-β/SMAD signaling activation.^196^ GLRX1 inhibits the phosphorylation of SMAD3 by deglutathionylation, resulting in the suppression of fibrotic gene expression and HSC activation.^197^ SUMOylation of FXR decreases its activity, promoting HSC activation and aggravating liver fibrosis.^198^

HSC activation is not an autonomous process but is heavily influenced by paracrine signaling from other liver cells, including hepatocytes, LSECs, and immune cells. Conversely, activated HSCs secrete various factors that further shape the fibrotic microenvironment. For example, activated HSC-derived exosomes can induce glycolysis in quiescent HSCs, immune cells, and LSECs by delivering glycolysis-related proteins, which further accelerate liver fibrosis.^199^ Specifically, exosomal DHFR derived from HSCs promotes M1 macrophage polarization and plays a crucial role in the crosstalk between HSC activation and inflammatory responses. A subset of HSCs overexpressing periostin secretes bone morphogenetic protein-1, which induces EMT in hepatocytes by activating EGFR signaling and contributes to liver fibrosis.^200^ Conversely, the LIM homeobox gene Lhx2, which is expressed specifically in HSCs, suppresses fibrogenesis and upregulates hepatocyte growth factor, which is crucial for liver regeneration.^201^ Activated HSCs also alter hepatocyte metabolism by increasing IGFBP3 and decreasing SERPINA12 secretion, leading to lipid droplet accumulation and lipogenic gene activation.^202^ This bidirectional crosstalk creates a self-reinforcing feedback loop, perpetuating liver fibrosis.

Notably, conventional wisdom has long regarded HSCs as the primary culprits of liver fibrosis, with some studies even advocating their direct ablation as a therapeutic strategy. However, recent investigations revealed that HSC depletion changed the zonation of hepatocytes, leading to marked alterations in liver regeneration, cytochrome P450 metabolism, and injury via R-spondin 3.^171^ Another study revealed that HSCs critically regulate hepatic energy substrate preference during fasting through PLVAP-mediated metabolic switching from fatty acid oxidation to carbohydrate utilization.^203^ These findings challenge the traditional therapeutic paradigm of HSC eradication. Instead, pharmacological reprogramming of activated HSCs to regain their quiescent phenotype represents a more physiologically sound and therapeutically advantageous strategy for combating liver fibrosis, as it preserves the essential homeostatic functions of these multifaceted liver sentinels.

#### Role of liver sinusoidal endothelial cells in liver fibrosis

LSECs are highly specialized liver-specific endothelial cells distinguished by their exceptional permeability, which stems from their unique structural features—fenestration lacking diaphragms and the absence of a basement membrane. Positioned at the blood‒liver interface, LSECs play a critical role in regulating material exchange and maintaining hepatic homeostasis.^204^ However, during liver fibrosis, LSECs undergo pathological capillarization, which is characterized by the loss of fenestrae, deposition of a basement membrane, and upregulation of capillarization-associated markers. This process severely compromises their permeability, filtration, and clearance functions.^205^ Notably, LSECs are among the earliest cells to sustain injury in fibrogenesis, indicating their pivotal role in initiating the fibrotic cascade. Its contribution to liver fibrosis progression is mediated through four key mechanisms: sinusoid capillarization, dysregulated angiogenesis, aberrant angiocrine signaling, and increased vasoconstriction.^206^ These pathological changes collectively drive HSC activation, macrophage recruitment, and hepatocyte injury, perpetuating fibrogenesis.

Recent advances in single-cell transcriptomics have revealed the spatial zonation-dependent heterogeneity of LSECs and their distinct roles in liver fibrosis. Three functionally specialized LSEC subpopulations have been identified: pericentral LSECs (expressing *Rspo3*, *Wnt9b*, *Kit*, *Cdh13*, *Thbd*, and *Fabp4*), midzonal LSECs (expressing *Lyve1* and *Cts1*), and periportal LSECs (expressing *Dll4*, *Efnb2*, *Msr1*, *Ltbp4*, *Ntn4*, and *Adam23*). Among these, pericentral LSECs exhibit the most pronounced upregulation of capillarization-related genes during fibrosis, indicating their central role in disease progression.^207^ These cells secrete CXCL9, a potent chemokine that drives macrophage chemotaxis and accelerates fibrogenesis.^208^ Additionally, fibrotic scars exhibit expansion of *ACKR1*^+^ and *PLVAP*^+^ LSEC subpopulations, which enhances leucocyte transmigration and exacerbates inflammation.

LSECs engage in extensive crosstalk with other liver cells to further amplify fibrotic progression.^209^ In a healthy liver, LSECs maintain HSC quiescence via NO secretion. During liver fibrosis, capillarized LSECs shift toward a profibrotic phenotype, secreting mediators such as fibronectin, fibrinogen, TGF-β, PDGF, endothelin, Hedgehog ligands, IL-1β, and A-FABP, all of which promote HSC activation.^210^ Recently, runt-related transcription factor 3 (RUNX3) deficiency in LSECs was shown to lead to dysfunction and increased production of leucine-rich alpha-2-glycoprotein 1, which activates HSCs in a paracrine manner.^211^ Furthermore, maladaptive crosstalk between histone deacetylase 2 and DNA methyltransferase 1 drives LSECs to produce fibrogenic EVs containing IGFBP7 and ADAMTS1, recruiting profibrotic Th17 cells to the liver.^212^ Epigenetic regulation also plays a critical role. The transcriptional coactivator p300 upregulates CCL2 expression in LSECs through acetylation of H3K27 in the CCL2 enhancer and promoter regions, as well as through its interaction with NF-κB and BRD4. The subsequent secretion of CCL2 serves as an angiocrine signal that promotes the recruitment of monocytes and macrophages, exacerbating inflammatory and fibrotic responses.^213^ Additionally, capillarization of LSECs induces a hypoxic microenvironment by impairing oxygen diffusion, which independently exacerbates HSC activation.^214^ Mechanotransduction-induced glycolysis in LSECs increases the expression of CXCL1 via NF-κB and epigenetic modifications at H3K27, driving neutrophil migration, portal hypertension, and accelerated fibrosis.^215^ Interestingly, LSECs play a dual role in liver fibrosis—while they predominantly drive fibrogenesis, they also regulate liver regeneration. In addition to profibrotic signaling, LSECs can induce hepatocyte growth factor (HGF) and Wnt2 while inhibiting NADPH oxidase 4 (NOX4) secretion, thereby promoting hepatocyte proliferation and repair to alleviate liver fibrosis.^90^ Targeting the balance between pro-regenerative and pro-fibrotic angiocrine pathways in LSECs may present an attractive strategy to achieve hepatic recovery while alleviating fibrosis.

#### Role of immune cells in liver fibrosis

Immune cells, including neutrophils, T cells, innate lymphoid cells, and macrophages, play critical roles in the pathogenesis of liver fibrosis. Macrophages, including bone marrow-derived macrophages (BMDMs) and liver-resident Kupffer cells, are the most abundant immune cells and are important in the pathogenesis of liver fibrosis because of the production of TGF-β. Notably, BMDMs—rather than Kupffer cells—are the major source of TGF-β, driving HSC activation and ECM deposition.^216^ In addition, stiffness-dependent efferocytosis by macrophages facilitates the resolution of inflammation and fibrosis.^217^ Recent advances in single-cell and spatial transcriptomics have revealed remarkable heterogeneity among hepatic macrophages, challenging the traditional M1/M2 dichotomy. In healthy livers, macrophages are spatially segregated into two clusters: *CD74*^+^*CD5L*^-^ macrophages, which are located mainly in the periportal area and participate in the immune response and antigen presentation, and *CD74*^+^*CD5L*^+^ Kupffer cells, which are scattered through the lobules and are responsible for hepatic homeostasis.^173^ During fibrosis, monocytes infiltrate the liver and differentiate into Ly-6C^+^ macrophages, which are recruited by CCL2 secreted from HSCs. These proinflammatory macrophages exacerbate fibrosis through TGF-β/PDGF-mediated HSC activation.^218^ TREM2^+^CD9^+^ scar-associated macrophages derived from circulating monocytes exhibit strong profibrogenic activity.^219^ Thbs1^+^ macrophages promote HSC activation via the PI3K/AKT/mTOR pathway.^220^

In addition to macrophages, neutrophils influence liver fibrosis through the formation of neutrophil extracellular traps, which promote HSC activation, proliferation, and migration via metabolic reprogramming, which is dependent on Nod-like receptor protein 3 activation.^191^ However, paradoxically, neutrophil depletion during the resolution phase has been associated with persistent hepatic inflammation, sustained fibrogenic activity and early fibrosis, suggesting a protective role for neutrophils in fibrosis resolution.^221^ T cells and innate lymphoid cells (ILCs) exert dual effects on liver fibrosis. They modulate HSC activation through inflammatory cytokines and chemokines while simultaneously influencing immune responses via feedback mechanisms.

In addition to their classical roles, immune cells regulate the fate of hepatocytes, LSCEs, and HSCs. Macrophages directly activate HSCs by secreting a variety of cytokines and chemokines, such as TGF-β, PDGF, IL-1β, TNF, CCL2, and CCL5.^21^ During fibrosis resolution, Ly6C^low^ macrophages (derived from Ly6C^high^ precursors) promote collagen degradation, hepatocyte proliferation, and HSC apoptosis through MMPs and TRAIL.^222^ Neutrophil-derived miR-223-enriched EVs inhibit HSC activation via Gli2 signaling. When taken up by hepatocytes, these EVs downregulate TAZ, reducing Indian hedgehog (IHH) release and preventing excessive HSC activation.^223^ M1 macrophage-conditioned medium enhances HSC proliferation, migration, and contractility.^222^ CD27^+^CD11b^+^ double-positive NK cells activate HSCs via ITGA4-VCAM1 binding with HSCs.^224^ In contrast, TIM4^+^ macrophages can enhance the clearance of apoptotic hepatocytes and inhibit HSC profibrotic activation by releasing IL-10.^225^

### Renal fibrosis

#### Epidemiology and etiology

Renal fibrosis, the hallmark of CKD, affects nearly 10% of the global adult population and is characterized by the progressive loss of renal function.^226^ Renal fibrosis may ultimately develop into end-stage renal disease (ESRD) and necessitate life-sustaining interventions such as long-term dialysis or kidney transplantation to prevent mortality. Approximately 800 million individuals suffer from CKD globally, with its prevalence and incidence steadily increasing, posing a considerable challenge to clinical treatment.^227^ The etiology of CKD is multifactorial, with major risk factors including aging, diabetes mellitus, hypertension, and obesity.

**Fig. 6:**
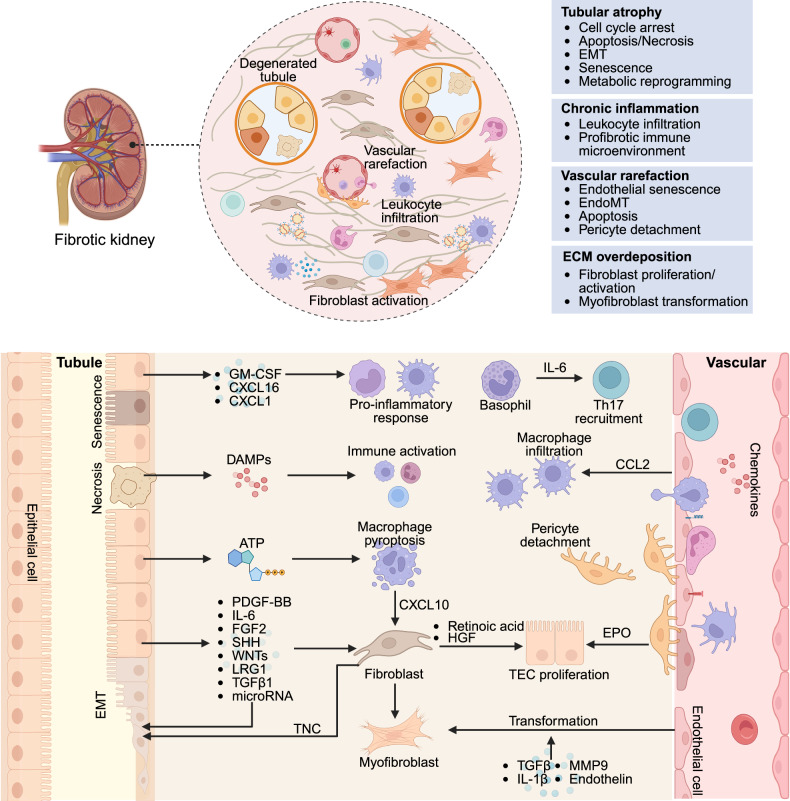
Mechanisms and cellular interactions in renal fibrosis. In fibrosis, tubular epithelial cells undergo cell cycle arrest, metabolic remodeling, senescence, and identity loss due to cell death and EMT, leading to tubular atrophy. Additionally, vascular rarefaction occurs from endothelial cell dysfunction, alongside chronic inflammation. Extensive leukocyte infiltration and immune microenvironment shifts contribute to excessive ECM deposition. Injured epithelial and endothelial cells release DAMPs and chemokines, inducing proinflammatory responses, immune cell infiltration, and effector molecules that activate fibroblasts. Fibroblasts reciprocally induce EMT. Notably, fibroblasts also secrete proregenerative effectors such as retinoic acid and HGF, promoting tubular epithelial cell (TEC) proliferation. DAMPs damage-associated molecular patterns, EMT epithelial‒mesenchymal transition, EPO erythropoietin, HGF hepatocyte growth factor, GM-CSF granulocyte‒macrophage colony‒stimulating factor, PDGF-BB platelet‒derived growth factor-BB, FGF2 fibroblast growth factor 2, SHH sonic hedgehog, LRG1 leucine‒rich α-2 glycoprotein 1, TNC tenascin-C, ATP adenosine 5’-triphosphate. This tool was created with BioRender (https://www.biorender.com/)

Renal fibrosis is a highly dynamic and complex pathological process driven by a cascade of interrelated cellular and molecular events, including kidney cell injury (e.g., tubular epithelial cell senescence, cell cycle arrest, and apoptosis), massive infiltration of inflammatory cells (e.g., granulocytes, macrophages, and T/B lymphocytes), activation of fibroblasts/myofibroblasts, vascular rarefaction (e.g., endothelial senescence and apoptosis), and tubular atrophy (Fig. 6).^7^ The progression of renal fibrosis is concomitant with a progressive decline in kidney function, manifested by reduced renal blood flow and tissue hypoperfusion, a decreased glomerular filtration rate, impaired tubular reabsorption of water and electrolytes, and elevated urinary protein excretion.^228^ The most widely adopted clinical diagnostic criteria include an estimated glomerular filtration rate (eGFR) < 60 mL/min/1.73 m² or a urinary albumin-to-creatinine ratio (ACR) ≥ 30 mg/g.^229^ Despite substantial advances in understanding renal fibrosis, key molecular mechanisms remain incompletely elucidated, and no effective antifibrotic therapies currently exist to halt or reverse disease progression. Systematic investigations of the interconnected regulatory networks among tubular epithelial cells, vascular endothelial cells, pericytes, activated fibroblasts, infiltrating immune cells, ECM components, secreted soluble mediators, and extracellular vesicles within the renal fibrogenic niche may reveal novel therapeutic targets.

#### Role of tubular epithelial cells in renal fibrosis

Renal tubular epithelial cells (TECs), the fundamental functional units of the nephron, play a critical role in maintaining renal homeostasis. Owing to their high mitochondrial density and reliance on oxidative phosphorylation for energy production, TECs are particularly vulnerable to various pathological insults, such as obstruction, hypoxia, and ischemia.^230^ Under physiological conditions, TECs remain quiescent and do not undergo active mitosis. However, in response to injury, they display remarkable plasticity. Following mild or transient damage, surviving TECs rapidly initiate an adaptive tubular repair process involving dedifferentiation, migration, proliferation, and subsequent redifferentiation to replace damaged cells, ultimately restoring renal structure and function. In contrast, severe or sustained injury triggers maladaptive remodeling in TECs, leading to irreversible tubular atrophy, chronic inflammation, and fibrosis.

Recent studies have elucidated the dynamic phenotypic and functional changes in TECs under stress, providing new insights into their regulatory role in renal fibrosis. In chronic aristolochic acid nephropathy, a fibrosis-related Havcr1^+^Chd2^+^ proximal tubular cell (PTC) subset termed “dedifferentiated senescent PTC” was identified. This subset showed increased enrichment of pathways related to cell growth and death, cellular senescence, and immune system activation.^231^ scRNA-seq analysis of UUO kidneys revealed a specific PTC subpopulation expressing elevated *Pdgfb*, a key secretory molecule involved in fibroblast activation, alongside proinflammatory gene signatures (e.g., *Cd74*, *Tnfrsf12a*, *Cxcl1*, *Cxcl10*, *Cxcl16*). Further receptor‒ligand interaction analysis indicated that this PTC subpopulation recruits myeloid and lymphoid cells while promoting localized fibroblast activation.^232^ Moreover, metabolic reprogramming was observed in PTCs from both uni-IRI and UUO kidneys.^233^ In the uni-IRI model, PTC injury was accompanied by abnormal lipid metabolism, whereas UUO induced aberrant amino acid metabolism.^233^ This highlights the value of integrating single-cell transcriptomic and metabolomic data to decipher the adaptive and maladaptive responses of TECs to injury.

Persistent DNA damage is a fundamental event in chronic kidney disease progression. Deficiency in the DNA repair protein Fan1 in TECs results in unresolved DNA damage, triggering epithelial polyploidization and renal tubular injury.^234^ In addition, epigenetic modifications play crucial roles in regulating TEC functions and influencing renal fibrosis. Aberrant m^6^A methylation in renal TECs can shift cellular functions toward a profibrotic phenotype. For example, upregulation of the m^6^A methyltransferase METTL3 in the epithelial cells of mice and human patients with renal fibrosis enhances m^6^A modification of *NET* mRNA, thereby increasing its stability and protein expression, which promotes the transcription of profibrotic genes in epithelial cells.^235^

A hallmark of renal fibrosis is the extensive loss of TECs. Unlike apoptosis, regulated necrosis, including ferroptosis, pyroptosis, and necroptosis, triggers inflammatory cascades and fibroblast activation via the release of cellular contents/cytokines.^230^ For example, HMGB1 release from pyroptotic TECs amplifies inflammation by activating caspase-11 and IL-1β in macrophages.^236^ Necroptotic TECs undergo plasma membrane rupture, releasing damage-associated molecular patterns (DAMPs) and cytokines to activate DCs and prime T cells, thereby driving the adaptive immune system and exacerbating kidney injury.^230^ TECs undergoing ferroptosis can release profibrotic factors, including PDGF-BB and IL-6, to trigger fibroblast-to-myofibroblast transition and induce EMT in adjacent TECs.^237^ In addition, persistent autophagy in injured TECs leads to fibroblast activation through the secretion of the paracrine factor FGF2.^238^

Following injury, TECs release metabolites, cytokines, and exosomes carrying cargo that mediate intercellular communication and amplify fibrotic signaling. TEC-released ATP can induce macrophage pyroptosis, leading to CXCL10 production and fibroblast activation.^239^ GM-CSF from TECs stimulates the CCR2/MCP-1 signaling pathway to promote proinflammatory cell infiltration and myofibroblast activation.^240^ TEC-derived CXCL16 recruits CXCR6^+^ monocytes/macrophages from the bone marrow, exacerbating UUO-induced renal fibrosis.^241^ Leukocyte-derived TNF further induces epithelial cells to release IHH, thereby driving the proliferation of GLI^+^ cells.^242^ TECs also secrete profibrotic cytokines, including SHH, WNTs, LRG1, and TGF-β1, to regulate fibroblast activation.^7,243^ Moreover, tubular cell-derived exosomal miR-150-5p and miR-21 can activate fibroblasts to contribute to renal fibrosis.^244^ During the progression of chronic kidney disease, the remodeling of pyruvate metabolism in renal TECs leads to substantial lactate accumulation. Elevated lactate levels in the fibrotic niche promote macrophage polarization toward the M2 phenotype by increasing H4K12 lactylation, thereby exacerbating fibrosis.^245^ Furthermore, TEC-produced lactate promotes histone acetylation in these cells, leading to increased production and release of TGF-β1, which in turn activates the SMAD3 signaling pathway in macrophages to trigger MMT.^246^

#### Role of fibroblasts in renal fibrosis

Activated myofibroblasts mainly promote scar formation by generating excessive ECM. However, their cellular origins remain a subject of ongoing debate. Perivascular cells constitute the primary source of myofibroblasts, whereas other cell types, including epithelial cells, endothelial cells, and bone marrow-derived cells, may also serve as progenitor cells for myofibroblasts via epithelial-to-mesenchymal transition (EMT), endothelial-to-mesenchymal transition (EndoMT), and macrophage-to-mesenchymal transition (MMT) processes^247^ Identifying the origin and markers of ECM-producing myofibroblasts is critical for precisely targeting the differentiation process and eliminating pathogenic subsets. A comprehensive cell atlas of renal fibrosis in humans and mice revealed that massive amounts of extracellular matrix is derived from Pdgfra^+^/Pdgfrb^+^ dual-positive fibroblasts and myofibroblasts. These cells mainly originate from pericytes and fibroblasts, with a minor contribution from dedifferentiated PTCs. NKD2 is highly expressed in this population, suggesting its potential utility as a biomarker for profibrotic myofibroblasts and a possible therapeutic target.^248^

While perivascular cells and/or myofibroblasts are traditionally viewed as profibrotic effector cells, emerging evidence indicates that they also play reparative functions in kidney injury through paracrine signaling mechanisms. Perivascular cell-derived erythropoietin (EPO) binds EPO receptors expressed on TECs, promoting TEC regeneration.^249^ Myofibroblast-secreted factors, including retinoic acid and hepatocyte growth factor, stimulate tubular cell survival and proliferation.^247^ The EphrinB2‒EphB4 signaling axis between pericytes and endothelial cells plays a critical role in maintaining vasculature stabilization during kidney injury. Inhibition of EphrinB2 signaling exacerbates capillary rarefaction and renal fibrosis.^250^ Furthermore, activated fibroblasts can orchestrate a profibrotic niche by releasing tenascin-C (TNC) to promote fibroblast activation and trigger tubular EMT.^7^ Fibroblasts and myofibroblasts are central orchestrators of renal fibrosis, contributing not only to ECM accumulation but also to tubular repair and vascular homeostasis. A deeper understanding of their cellular origins, functional heterogeneity, and regulatory mechanisms will be essential for developing precision antifibrotic therapies.

#### Role of endothelial cells in renal fibrosis

As a highly vascularized organ, the kidney exhibits remarkable endothelial cell heterogeneity, particularly among vascular endothelial cells. These cells play multifaceted roles in addition to their conventional functions in oxygen and nutrient transport, coagulation regulation, and immune and inflammatory modulation, and they are also critical for maintaining and fine-tuning renal function. Renal vascular endothelial cells can be categorized into glomerular endothelial cells (GEnCs), peritubular capillary endothelial cells (PTECs), and endothelial cells lining larger veins and arteries.^251^ Structurally, arterial and venous endothelial cells are continuous, confluent, and elongated in the direction of blood flow. In contrast, GEnCs are highly fenestrated and coated with a thick luminal glycocalyx, which is crucial to glomerular filtration.^251^ PTECs also exhibit fenestrations, but their fenestrae are covered by thin diaphragms composed of glycoproteins, facilitating tubular secretion and reabsorption.^251^ However, at present, a comprehensive characterization of the xenogeneic EC population and its functions in fibrotic kidneys is lacking. A previous study using scRNA-seq analysis revealed *CD34*^+^*CDH5*^+^ high endothelial venules (HEVs) in the fibrotic microenvironment.^252^ Given the important role of CD34 in mediating intercellular adhesion, these *CD34*^+^*CDH5*^+^ HEVs may be involved in regulating the inflammatory milieu of renal fibrosis.

Progressive vascular rarefaction and leakage are hallmarks of renal fibrosis, driving tissue hypoxia and dysfunction in surrounding stromal and immune cells. A central mechanism underlying these pathological changes is EndoMT, which is promoted by profibrotic mediators in the tissue microenvironment, such as TGF-β, IL-1β, MMP-9, and endothelin.^253^ EndoMT, in turn, exacerbates metabolic shifts in TECs induced by tissue hypoxia (promoting glycolysis and inhibiting fatty acid oxidation) and enhances the inflammatory response.^254^ Interestingly, the lysine methyltransferase SET7 has been identified as a molecular switch regulating EndoMT, suggesting that targeting SET7 could mitigate capillary loss in renal fibrosis.^255^ Similarly, the inhibition of fibrillin-1 has demonstrated the potential to promote the proliferation of endothelial cells.^7^

Chronic exposure of endothelial cells to inflammatory cytokines (e.g., TNF, IFN-γ, and IL-1) and PAMPs induces intracellular signaling, which upregulates adhesion molecules (e.g., E-selectin, VCAM-1, and ICAM-1) to promote leukocyte recruitment while concurrently reducing the production of protective molecules such as thrombomodulin.^251^ Furthermore, injured endothelial cells also contribute to fibrosis by secreting CCL2 to promote macrophage infiltration and releasing LRG1 to activate TGF-β signaling in podocytes and mesangial cells.^256,257^ Additionally, increased glycolysis in fibrotic renal endothelial cells leads to the upregulation of the lactate exporter monocarboxylate transporter 4. The resulting accumulation of lactate in the extracellular matrix promotes monocyte/macrophage adhesion to the endothelium.^258^ These findings suggest that endothelial metabolic abnormalities can participate in fibrosis by regulating intercellular communication via metabolites.

#### Role of immune cells in renal fibrosis

During renal fibrosis, the immune response not only regulates the initiation and resolution of inflammation but also orchestrates interactions with stromal and parenchymal cells to drive tissue remodeling by establishing a profibrotic/antifibrotic immune microenvironment. Macrophages are central to renal immune surveillance and homeostasis. Upon injury, monocytes and macrophages can polarize and adopt specific phenotypes, including proinflammatory CD206^-^CD68^+^ M1 macrophages and anti-inflammatory, reparative CD206^+^CD68^+^ M2 macrophages.^241^ Emerging single-cell level analyses of fibrotic renal tissue have revealed that macrophage polarization is far more complex than the M1/M2 dichotomy is. Some macrophages coexpress both M1 and M2 markers,^259^ and their functional states are highly context dependent. This heterogeneity underscores the limitations of traditional classification systems and highlights the need for fibrosis-specific macrophage markers. Intriguingly, a recent study revealed a major profibrotic macrophage subset expressing Fn1, Spp1, and Arg1. Targeted depletion of these macrophages via bioactivated peptides has been shown to attenuate IRI-induced fibrosis by inducing macrophage death, reshaping the renal microenvironment, and inhibiting profibrotic immune responses. In addition, SPP1 produced by profibrotic macrophages was also found in patients, suggesting the therapeutic potential of targeting SPP1^+^ macrophages.^153^ Another study in a UUO model a profibrotic Ly6C^+^Arg1^+^ monocyte subset that promotes fibrosis through increased Fn1-integrin, Pdgfa-Pdgfrβ, and Tnf-Tnfsfr1 signaling to mesenchymal cells. Conversely, during the repair phase, monocytes differentiate into MMP12^+^ macrophages, which express scavenger receptors (*Mrc1*, *Fcrls*) and genes associated with efferocytosis and lipid transport. These cells are likely to promote tissue repair by degrading the ECM and clearing apoptotic cells.^260^

In addition to monocytes/macrophages, renal Tregs differentially expressed response genes in response to the inflammatory environment according to the results of scRNA-seq analysis. Fibrotic Tregs are enriched in inflammation- and apoptosis-related pathways, whereas regenerated Tregs are enriched in angiogenesis-related pathways.^261^ These results indicate that the underappreciated plasticity in Treg function within the same tissue has not been fully recognized thus far. Recent discoveries have expanded the spectrum of immune cells implicated in renal fibrosis. Upon tissue injury, CXCR6⁺ ILC3s in the intestinal mucosa rapidly respond to CXCL16 released by distally damaged tubular cells, leading to substantial ILC3 infiltration into the kidney. Within fibrotic kidneys, these ILC3s upregulate programmed cell death-1 (PD-1) expression and secrete IL-17A, directly activating myofibroblasts to produce excessive ECM.^262^ Furthermore, CXCR2^+^ basophils, which are recruited by profibrotic tubule-derived CXCL1, orchestrate renal fibrosis by promoting interleukin-6 release and subsequent Th17 recruitment.^232^ Although the immune microenvironment plays a pivotal role in regulating fibrosis, the environmental triggers that initiate its transition, as well as the key effector molecules within this microenvironment (e.g., metabolites, chemokines, and extracellular vesicles), are still poorly understood at a comprehensive level.

## Crucial signaling pathways involved in fibrosis

### TGF-β signaling pathway

**Fig. 7:**
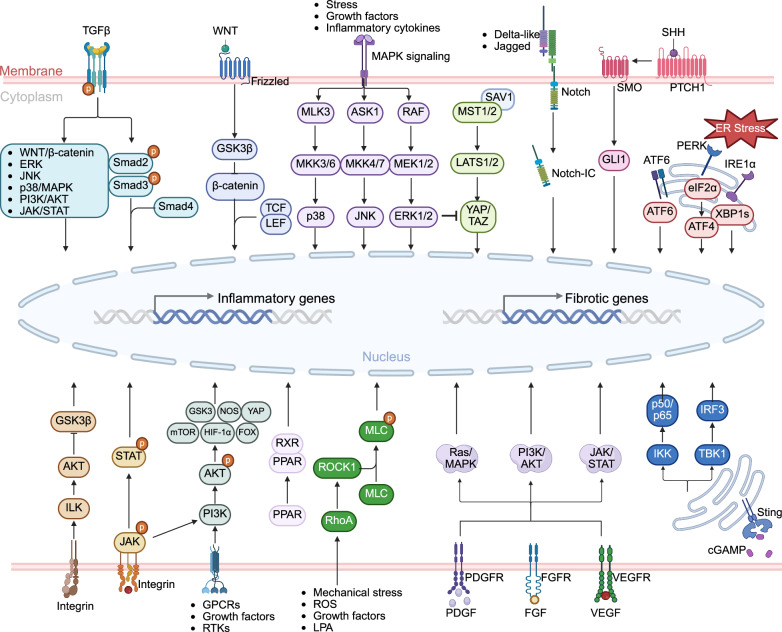
Schematic representation of critical signaling pathways involved in fibrosis. TGF-β transforming growth factor-β, ERK extracellular signal-regulated kinase, JNK c-Jun N-terminal kinase, MAPK mitogen-activated protein kinase, PI3K/AKT phosphatidylinositol 3-kinase/protein kinase B, JAK/STAT Janus kinase/signal transducer and activator of transcription, GSK3β glycogen synthase kinase 3β, MLK3 mixed-lineage kinase 3, ASK1 apoptosis signal-regulating kinase 1, MKK mitogen-activated protein kinase kinase, MEK mitogen-activated protein kinase, SAV1 salvador family WW domain containing protein 1, MST1/2 macrophage stimulating 1/2, LATS large tumor suppressor, YAP Yes-associated protein, TAZ transcriptional coactivator with PDZ-binding motif, SHH sonic hedgehog, PTCH1 protein patched homolog 1, SMO smoothened, GLI1 glioma-associated homolog 1, ER endoplasmic reticulum, ATF6 activating transcription factor 6, PERK protein kinase RNA-like endoplasmic reticulum kinase, eIF2α eukaryotic initiation factor 2 alpha, ATF4 activating transcription factor 4, IRE1α, inositol-requiring kinase 1alpha. This tool was created with BioRender (https://www.biorender.com/)

TGF-β is a multifunctional cytokine and major mediator of fibrosis (Fig. 7). TGF-β is initially synthesized as a precursor polypeptide consisting of three domains: an N-terminal signal peptide, a latency-associated peptide (LAP), and a C-terminal mature cytokine.^263^ Following translation, the precursor is translocated into the endoplasmic reticulum lumen, where it removes the signal peptide. The proprotein then traffics to the Golgi apparatus, where further proteolytic processing removes LAP and generates a mature TGF-β cytokine. Secreted TGF-β remains inactive due to LAP binding, forming a small latent complex that masks its receptor-binding site.^264^ ECM1 acts as a critical gatekeeper by stabilizing latent TGF-β,^265^ which requires activation by integrins, MMPs, ROS, and thrombospondin-1 (TSP-1).^266^ Once liberated from its latent form, bioactive TGF-β (three isoforms: TGF-β1/2/3) initiates fibrotic responses via canonical (SMAD-dependent) and/or noncanonical (SMAD-independent) pathways.

In the canonical pathway, TGF-β binds to the TGF-β receptor II (TβRII), activates TβRI, and then phosphorylates receptor-regulated SMADs (R-SMADs: SMAD2/3). R-Smad/Smad4 complexes then translocate to modulate gene expression.^267^ This cascade is negatively regulated by I-SMADs (SMAD6/7) and fine-tuned by posttranslational modifications or interactions with transcriptional cofactors.^268^ For example, SMAD acetylation enhances its phosphorylation, promotes its nuclear translocation, and amplifies TGF-β signaling.^196^

In addition to the SMAD-dependent pathway, TGF-β activates multiple noncanonical signaling cascades, including the MAPK (ERK, JNK, p38), PI3K/AKT, Wnt/β-catenin, JAK/STAT, and NF-κB signaling pathways.^269^ These pathways synergize with SMAD signaling to promote fibroblast-to-myofibroblast transition, ECM overproduction, and impaired degradation. Notably, TGF-β-induced NUAK1 amplifies fibrotic responses via YAP and TGF-β/Smad crosstalk, whereas Hippo effectors (YAP/TAZ) stabilize SMAD2 to exacerbate fibrosis.^270^ Notch inhibition attenuates fibrogenesis by suppressing TGF-β/SMAD2 activation.^271^ As the primary fibrogenic hub, TGF-β interacts with nearly all fibrosis-related pathways. Therapeutic strategies targeting TGF-β activation or downstream signaling show promise in mitigating organic fibrosis.

### Wnt signaling pathway

The Wnt signaling pathway comprises canonical (β-catenin dependent) and noncanonical (β-catenin independent) pathways, both of which are implicated in fibrosis across multiple organs. In the canonical pathway, Wnt ligands bind the FZD receptor and LRP5/6 coreceptors, triggering LRP5/6 phosphorylation and DVL recruitment. This disrupts the β-catenin destruction complex (GSK3β, CK1α, AXIN, and APC), stabilizing β-catenin for nuclear translocation, where it binds TCF/LEF to drive fibrotic gene expression.^272^

In liver fibrosis, β-catenin inhibition restrains bile acid synthesis and suppresses HSC activation and collagen synthesis, whereas enhancing HSC glycolysis exacerbates fibrosis.^273^ In IPF, aberrant Wnt/β-catenin activation worsens fibrosis, whereas its inhibition alleviates disease progression. In cardiac fibrosis, Wnt/β-catenin activates cardiac fibroblasts, particularly endocardium-derived fibroblasts, a key population in cardiac remodeling.^274^ In age-related renal fibrosis, Wnt/β-catenin upregulates AT1 receptors, induces mitochondrial dysfunction, and, via Wnt9a, drives tubular senescence and TGF-β1-mediated renal fibroblast activation.^275^ Specifically, the activation of Wnt/β-catenin signaling in macrophages promotes M2 polarization to promote kidney fibrosis.^276^

In addition to the canonical Wnt pathway, noncanonical pathways also contribute to fibrosis. During organ fibrosis, elevated ligands (Wnt5a, Wnt4, and Wnt11) contribute to fibrosis via FZD5‒EGFR crosstalk, with Wnt5a/Wnt11 inhibition ameliorating cardiac fibrosis.^277^ Noncanonical Wnt5a also controls the activation of latent TGF-β to drive fibroblast activation and tissue fibrosis.^278^ Additionally, Wnt signaling can interact with multiple pathways to amplify fibrosis. TGF-β induces long noncoding RNA00313 to activate Wnt signaling^279^ and upregulates SFRP2 to modulate noncanonical Wnt.^277^ Hedgehog/Gli signaling induces Wnt10a to promote myofibroblast activation. Wnt/β-catenin can sustain Notch signaling via Jagged1/Jag2b upregulation.^280^ YAP-targeted Wntless enhances noncanonical Wnt and β-catenin.^281^

### MAPK signaling pathway

The mitogen-activated protein kinase (MAPK) signaling pathway is a critical mediator that modulates cell proliferation, differentiation, and death. This pathway comprises three core kinases—MAP3K, MAPKK, and MAPK—and includes three major subfamilies: JNK, ERK, and p38.^282^ Upon activation by extracellular stimuli (e.g., hormones, cytokines, and growth factors), MAPK phosphorylates and activates downstream effectors to modulate target gene expression,^283^ thereby driving fibroblast proliferation, activation, and ECM production in organ fibrosis.

JNK promotes fibrotic gene expression via phosphorylation of c-Jun. Overactivation of JNK1/2 accelerates fibroblast proliferation and fibrosis progression. A selective JNK inhibitor (CC-930, CC-90001) reduces MMP7 levels and has potential in IPF.^284^ JNK also cross-talks with other pathways to amplify organ fibrosis. In HSCs, JNK enhances TGF-β1 transactivation and initiates SMAD3 phosphorylation, which subsequently promotes ECM production and exacerbates liver fibrosis.^285^ In renal fibrosis, JNK activation augments TGF-β transcription, activates latent TGF-β, and directly phosphorylates SMAD3. Wnt5a-induced JNK activation and the AKT-JNK interplay further accelerate fibrosis.^286^ DUSP1 attenuates fibrosis by suppressing cGAS-STING signaling via JNK dephosphorylation.^287^

ERK drives fibrosis mainly through ECM accumulation. Hyperphosphorylated ERK promotes EMT, fibroblast proliferation and activation, and ECM deposition. ERK activation can induce mitochondrial fragmentation and ROS overexpression, which further worsens IPF. ERK inhibition mitigates renal fibrosis by suppressing early growth response 1 and FGF2.^288^ IL-11 is a crucial determinant of organ fibrosis, and its effect is mediated by ERK activation, which is often modulated by TGF-β, AKT, and YAP.^289^

Activated by stress and inflammatory cues, p38 signaling regulates fibrosis via inflammation, myofibroblast differentiation, and ECM deposition.^17^ A recent study suggested that p38 signaling plays a vital role in regulating HSC activation via the YAP signaling pathway.^290^ p38 activation promotes NLRP3-mediated hepatocyte pyroptosis, activating HSCs and fueling liver fibrosis.^291^ p38 inhibition attenuates TGF-β and collagen-1 overexpression in lung injury and cardiac remodeling. In renal interstitial fibrosis, p38 suppression downregulates autophagy and fibrosis via TGF-β/SMAD signaling.

### Hippo/YAP signaling pathway

The Hippo signaling pathway, a conserved kinase cascade, critically regulates the nuclear translocation of the transcriptional coactivators YAP and TAZ, serving as pivotal modulators of fibrotic progression across multiple organs.^292^ Dysregulation of this pathway disrupts tissue homeostasis, driving fibroblast activation, aberrant ECM remodeling, and sustained fibrogenesis through context-dependent activation mechanisms and crosstalk with diverse signaling pathways.^293^

In cardiac fibrosis, transient ERBB2 activation induces ERK-dependent YAP phosphorylation and mechanotransduction signaling, triggering EMT-like cytoskeletal remodeling;^294^ conversely, pathological mechanical stress postmyocardial injury drives nuclear YAP/TAZ-TEAD activation, fostering myofibroblast differentiation.^295^ In hepatic fibrosis, quiescent HSCs undergo YAP-dependent activation into myofibroblastic HSCs (mf-HSCs), a key driver of fibrogenesis. Hedgehog-YAP-regulated glutaminolysis sustains mf-HSCs metabolic reprogramming,^296^ whereas ischemia‒reperfusion injury paradoxically activates hepatocyte YAP to attenuate oxidative stress yet suppresses HSC activation, suggesting its dual role.^297^ Early fibrogenesis involves Hippo–YAP-mediated upregulation of *Ctgf* and *Ankrd1*, whereas pharmacological YAP inhibition (e.g., verteporfin) suppresses fibrosis.^298^ Macrophage-specific YAP amplifies profibrotic CTGF^+^VCAM1^+^ endothelial signaling via suppressed type I interferon responses.^225^ Notably, HIV exacerbates liver fibrosis via LPAR1/PI3K/AKT-mediated YAP activation.^299^ In systemic sclerosis, YAP/TAZ-TEAD synergizes with TGF-β/Wnt to induce myofibroblast differentiation and EndoMT, which is reversible by TEAD inhibition.^300^ Single-cell sequencing and spatial transcriptomics implicate SFRP4^+^ and CXCL13^+^ fibroblasts as Hippo-driven ECM producers.^301^ These new technologies provide a comprehensive perspective for understanding HS pathogenesis. In pulmonary fibrosis, Hippo/Yap inactivation promotes Taz-mediated AT1 cell differentiation, whereas YAP-MYC cooperation drives bronchiolization and fibrosis in murine injury models.^302^

### JAK/STAT signaling pathway

The JAK-STAT pathway constitutes an evolutionarily conserved signaling cascade comprising four Janus kinases (JAK1, JAK2, JAK3, TYK2) and seven STAT transcription factors (STAT1-4, STAT5A, STAT5B, STAT6).^303^ Upon cytokine or growth factor stimulation, membrane-associated JAKs undergo activation and subsequently phosphorylate STAT proteins, facilitating their dimerization and nuclear translocation to modulate gene expression. This pathway critically regulates cellular proliferation, metabolic homeostasis, and immune responses.^303^ Dysregulated JAK-STAT signaling contributes to fibrogenesis through sustained inflammation, aberrant ECM deposition, and progressive organ dysfunction.^304^

Bone marrow-derived mesenchymal stromal cells (BM-MSCs) in myeloproliferative neoplasm (MPN) patients drive fibrosis via JAK1/2-STAT3 and TGFβ-SMAD3 crosstalk.^305^ In myelofibrosis, JAK inhibitors attenuate signal transduction, mitigating inflammation and fibrosis. T-cell protein tyrosine phosphatase (TCPTP) acts as a key negative regulator of the JAK/STAT signaling pathway by dephosphorylating and inactivating JAK-1 and JAK-3. In liver fibrosis, TCPTP inactivation elevates hepatic STAT-1/STAT-3 signaling, driving T-cell recruitment, MASH, and fibrosis via STAT-1, while STAT-3 independently promotes HCC through oncogenic autocrine loops, dissociating fibrosis from tumorigenesis.^306^ In UUO and IRI mouse models, TREM2 deficiency activates JAK2-STAT1/STAT3 signaling to promote M1/M2 macrophage polarization, leading to increased apoptosis and inflammation and exacerbated renal fibrosis.^307^ In murine skin fibrosis models, SHP2 ablation attenuates TGF-β-induced JAK2/STAT3 activation by increasing JAK2 phosphorylation at Y570, mitigating fibroblast activation and dermal fibrosis.^308^ Despite these advances, the cell type-specific roles of JAK-STAT signaling in the pathogenesis of fibrosis remain incompletely characterized. Future studies should integrate high-throughput transcriptomics to delineate the spatiotemporal regulation of this pathway.

### PI3K/AKT signaling pathway

The PI3K/AKT signaling pathway is a central intracellular signaling cascade that governs critical cellular processes, including cell growth, proliferation, motility, metabolism, and survival. PI3K consists of a catalytic domain (p110) and a regulatory domain (p85), which are activated by receptor tyrosine kinases (RTKs), G-protein-coupled receptors (GPCRs), and growth factors (GFs).^309^ Upon activation, PI3K phosphorylates phosphatidylinositol 4,5-bisphosphatе (PIP2) to gеnеratе phosphatidylinositol 3,4,5-trisphosphatе (PIP3), a second messenger that recruits AKT to the plasma membrane. AKT is then phosphorylated and activated, subsequently modulating downstream effectors to regulate diverse biological responses.

In IPF, hyperactivation of the PI3K/AKT pathway drives excessive α-SMA, a hallmark of myofibroblast activation and ECM deposition.^310^ Crosstalk with TGF-β signaling further amplifies fibrotic responses, promoting fibroblast proliferation and collagen synthesis. Additionally, the activation of PI3K/AKT participates in lung fibrosis by regulating its downstream mediators, including mTOR, HIF-1α, and the FOX family. In cardiac fibrosis, PI3K/AKT signaling influences myocardial remodeling by regulating cell survival, apoptosis, and cardiac contractility through key effectors such as mTOR, GSK-3, FoxO1/3, and nitric oxide synthase.^311^ In addition, the PI3K/AKT axis contributes to renal fibrogenesis by orchestrating inflammation, oxidative stress, apoptosis, autophagy, and EMT.^312^ Persistent AKT activation exacerbates tubulointerstitial fibrosis, highlighting its potential as a therapeutic target in chronic kidney disease.

### PPAR signaling pathway

Peroxisome proliferator-activated receptors (PPARs) belong to the nuclear hormone receptor superfamily and function as ligand-activated transcription factors that modulate gene expression in metabolic and fibrotic pathways. Structurally, PPARs form heterodimers with the retinoid X receptor upon ligand binding, enabling their translocation to the nucleus and subsequent binding to peroxisome proliferator response elements (PPREs) in target gene promoters. The PPAR family comprises three isoforms with distinct but overlapping functions: PPARα—which primarily regulates lipid metabolism and inflammation; PPARβ/δ—which modulates wound healing and metabolic adaptation; and PPARγ—which is critical for adipocyte differentiation and antifibrotic responses.^313^ The activation of PPAR signaling pathways is essential for reversing fibrogenic processes. For example, PPARα agonists inhibit HSC activation and collagen production, attenuating liver, kidney, and cardiac fibrosis in animal models.^314^ In bleomycin-induced pulmonary fibrosis, PPARα activation significantly reduces the fibrotic burden, whereas PPARα-knockout mice exhibit exacerbated fibrosis.^315^ PPARβ/δ serves as a key regulator of the inflammation-to-repair transition, preventing excessive fibrogenesis in various fibrosis models; its activation promotes macrophage polarization toward an antifibrotic phenotype, facilitating tissue repair.^316^ PPARγ ligands and agonists can antagonize TGF-β-driven fibrogenesis by suppressing myofibroblast differentiation and NF-κB-mediated inflammation, demonstrating effectiveness in alleviating liver, lung, cardiac, and renal fibrosis in preclinical models.

### Notch signaling pathway

The Notch signaling pathway is a phylogenetically conserved system comprising cell-surface Notch receptors and their cognate ligands. Ligand‒receptor interactions trigger γ-secretase-mediated proteolytic cleavage, releasing the Notch intracellular domain (NICD), which translocates to the nucleus to activate downstream targets. This pathway fundamentally regulates cell fate determination, proliferation, and survival and participates in context-dependent fibrotic processes.

Notch signaling exacerbates renal fibrosis by enhancing CCR2-dependent recruitment and activation of bone marrow-derived macrophages, thereby promoting TGF-β secretion and EMT.^317^ Epigenetic regulation further sustains Notch-driven fibrosis, as DNA methylation of the *HOXA5* promoter inhibits its expression, thereby transcriptionally repressing *JAG1* and activating Jag1-Notch signaling.^318^ In murine liver fibrosis models, endothelial POFUT1 deficiency increases fibrinogen, exacerbating LSEC dysfunction and HSC activation.^319^ Hepatocyte-specific Notch activation induces Sox9-dependent osteopontin secretion, fueling HSC activation and MASH-associated fibrogenesis; therapeutic targeting of osteopontin-mediated profibrotic crosstalk may mitigate fibrosis while circumventing the systemic toxicity of pan-Notch inhibition.^320^ Additionally, hepatocyte-derived JAG1 drives MASH-induced fibrosis via TLR4-NF-κB-mediated Notch activation in pericentral hepatocytes, which promotes fibrogenic pathways such as Spp1. EphB2, a Notch-induced effector in hepatocytes, promotes MASH-associated fibrosis through cell-autonomous inflammation and intercellular communication with nonparenchymal cells.^321^ Furthermore, lnc-LFAR1 binds SMAD2/3 and promotes TGFβR1 interaction and SMAD2/3 phosphorylation, thereby establishing a profibrotic TGFβ/SMAD/Notch feedback loop in CCl_4_-induced liver fibrosis.^322^ Notch signaling modulates fibrotic responses in pulmonary injury by regulating immune cell polarization. Notch blockade increases the number of anti-inflammatory L4-type macrophages differentiated from circulating monocytes, attenuating fibrosis in bleomycin-induced and SARS-CoV-2-infected models.^323^ In IPF, Notch1 hyperactivation in alveolar epithelial cells impairs Napsin A activity and SP-B processing, promoting AT2 cell proliferation, dedifferentiation, and fibroproliferation.^324^ Additionally, CXCR7 suppression in pulmonary capillary endothelial cells (PCECs) results in the recruitment of VEGFR1^+^ perivascular macrophages, which activate Wnt/β-catenin-dependent Jag1/Notch signaling in fibroblasts to drive fibrosis.^325^

### Integrin signaling pathway

Integrins are a family of transmembrane receptors that are composed of 24 distinct heterodimers formed through noncovalent interactions between 18 α-subunits and 8 β-subunits. These receptors play crucial roles in mediating cell‒matrix and cell‒cell interactions and the development of organ fibrosis. Integrins can be activated by a variety of external signals, including ECM components, mechanotransduction forces (e.g., stiffness and shear stress), and non-ECM ligands (e.g., GFRs, hormones, and small molecules).^326^ Once activated, integrins undergo clustering, leading to the expression of adaptor proteins such as talin and vinculin, which bridge integrins to the actin cytoskeleton. This “outside-in” signaling mechanism initiates multiple downstream pathways, including the integrin-linked kinase, focal adhesion kinase (FAK), Rho-associated coiled-coil kinase (Rho/ROCK), MAPK, and PI3K/AKT pathways. These pathways collectively regulate critical cellular processes, such as survival, proliferation, migration, polarity, and differentiation, all of which are involved in fibrosis progression. In addition to direct effects through ligand binding, integrins also interact with the arginine-glycine-aspartate (RGD) motif of latent TGF-β, promoting the release of active TGF-β and thereby exacerbating fibrosis through both canonical and noncanonical signaling pathways.^327^

### cGAS‒STING signaling pathway

Cyclic guanosine monophosphate-adenosine monophosphate synthase (cGAS) is a cytosolic DNA sensor that monitors pathogen infection or cellular stress by binding to double-stranded DNA (dsDNA) in the cytosol and converting ATP and GTP into 2′3′-cyclic GMP-AMP (cGAMP).^328^ As a second messenger, cGAMP subsequently binds to the adapter protein stimulator of interferon genes (STING) localized at the endoplasmic reticulum (ER) membrane. In the canonical pathway, STING translocates into the Golgi apparatus and activates TANK binding kinase 1 (TBK1) and IκB kinase (IKK). These kinases phosphorylate interferon regulatory factor 3 (IRF3) and NF-κB, respectively, leading to the transcriptional induction of type I interferons (IFNs) and several other inflammatory cytokines and chemokines.^329^ Alternatively, the noncanonical cGAS‒STING pathway operates independently of TBK1‒IRF3 and NF-κB activation. Instead, it is governed primarily by PKR-like endoplasmic reticulum kinase (PERK) and eukaryotic initiation factor 2α (eIF2α).^330^ Activated STING interacts with and directly activates PERK at the ER, promoting eIF2α phosphorylation and triggering diverse cellular responses, including autophagy, cellular senescence, and cell death.

Aberrant cGAS/STING activation has been implicated in inflammatory and fibrotic diseases.^330^ For example, liver and kidney fibrosis can be triggered by STING activation in response to cytosolic mitochondrial DNA (mtDNA) leakage, leading to chronic inflammation and tissue scarring.^331,332^ In lung fibrosis, cGAS activation by damaged autologous DNA promotes cellular senescence, exacerbating fibrotic progression.^333^ During myocardial infarction, cardiomyocyte-released dsDNA activates the cGAS-STING-IRF3 pathway in infiltrating macrophages, inducing cardiomyocyte apoptosis and fibroblast activation, ultimately worsening cardiac fibrosis and impairing heart function.^334^

### RhoA/ROCK signaling pathway

Rho-associated coiled-coil kinase (ROCK), a serine/threonine kinase regulated by the small GTPase RhoA, is involved in regulating cell contraction, polarity, adhesion, motility, proliferation, apoptosis, differentiation, maturation, and ECM deposition, all of which contribute to the progression of fibrosis.^335^ RhoA can be activated by diverse upstream stimuli, including ROS, GFs, lysophosphatidic acid (LPA), and ECM stiffening. Upon activation, Rho triggers ROCK-mediated signaling, leading to cytoskeletal reorganization (e.g., actin stress fiber formation), the assembly of focal adhesion complexes, and the transcriptional upregulation of profibrotic genes. In liver fibrosis, RhoA and its downstream effectors are highly expressed in hepatic vascular smooth muscle cells, vascular endothelial cells, and HSCs; their activation increases hepatic vascular resistance and exacerbates ECM deposition.^336^ Elevated levels of activated ROCK are observed in both patients with lung fibrosis and experimental animal models, while its pharmacological inhibition not only slows disease progression but also reverses established fibrosis.^337^ Additionally, in cardiac fibrosis, the RhoA/ROCK pathway is implicated in cardiac remodeling under pathological conditions such as hypertension, heart failure, cardiomyopathy, arrhythmia, and postmyocardial infarction repair.^338^ Persistent ROCK activation promotes excessive collagen deposition and myocardial stiffness. In the kidney, RhoA/ROCK signaling exacerbates fibrosis by inducing the expression of profibrotic mediators, including CTGF and TGF-β1, further perpetuating ECM accumulation and tissue scarring.^339^

### PDGF/PDGFR signaling pathway

The platelet-derived growth factor (PDGF) family is composed of five different disulfide-linked dimeric ligands (PDGF-AA/BB/AB/CC/DD), which exert their biological function by binding to two structurally related tyrosine kinase receptors, PDGFR-α and PDGFR-β.^340^ Upon ligand binding, PDGF receptors dimerize and undergo autophosphorylation, activating downstream signaling cascades such as the Ras/MAPK, PI3K/AKT, and JAK/STAT pathways.^340^ PDGF/PDGFR is involved in various pathophysiologic events, such as cell migration, division, growth, and proliferation. Numerous studies have highlighted the critical role of the PDGF/PDGFR axis in the pathogenesis of fibrosis across various organs. In liver fibrosis, elevated PDGFRs and their ligands have been observed in both experimental animal models and patients with liver fibrosis, suggesting a key role in HSC activation and ECM deposition.^341^ In renal fibrosis, PDGF promotes the proliferation of renal interstitial fibroblasts and their differentiation into myofibroblasts. Studies have demonstrated that anti-PDGFR-α/β antibodies attenuate fibrosis in UUO-induced renal fibrosis models.^342^ In cardiac and pulmonary fibrosis, PDGF contributes to fibrosis by activating fibroblasts and inducing their transformation into matrix-producing myofibroblasts.^343^

### FGF/FGFR signaling pathway

Fibroblast growth factors (FGFs) constitute a highly conserved family of 22 pleiotropic growth factors that regulate fundamental cellular processes, including migration, proliferation, differentiation, and survival. On the basis of their secretion patterns and mechanisms of action, FGFs are categorized into (1) the paracrine (subdivided into the FGF1, FGF4, FGF7, FGF8, and FGF9 subfamilies), (2) endocrine (FGF15/19, FGF21, and FGF23), and (3) intracrine (FGF11‒13) subgroups. Paracrine FGFs exhibit a high affinity for heparan sulfate, restricting their diffusion and enabling localized signaling, whereas endocrine FGFs demonstrate a reduced affinity for heparan sulfate, enabling their secretion into the general circulation to exert systemic endocrine effects in various organs.^344^

FGFs exert their biological effects by binding to four FGF receptors (FGFRs) with tyrosine kinase activity: FGFR1, FGFR2, FGFR3, and FGFR4.^345^ Paracrine FGFs require heparan sulfate proteoglycans for FGFR binding, inducing receptor dimerization and the activation of downstream pathways such as the Ras/MAPK, PI3K/AKT, and JAK/STAT signaling pathways. Endocrine FGFs necessitate Klotho proteins (alpha-Klotho or beta-Klotho) as coreceptors for FGFR activation. Paracrine FGFs can exert either antifibrotic or profibrotic effects during fibrogenesis.^344^ In pulmonary fibrosis, FGF1, FGF9, and FGF18 exhibit profibrotic effects, whereas FGF2, FGF7, and FGF10 demonstrate antifibrotic effects.^344^ FGF1 drives renal fibrosis and inflammation in immune-mediated injuries.^346^ Clinical observations revealed elevated FGF1/FGFR expression in fibrotic lesions of patients with lupus nephritis and acute interstitial nephritis, underscoring the clinical relevance of FGFs.^347^ Notably, FGF2 potentiates TGF-β1-mediated fibrotic responses, whereas FGF16 counteracts this effect. Endocrine FGFs such as FGF19 and FGF21 are gaining interest for their protective roles in multiple organs.^344,348^ Additionally, FGF23 has profibrotic effects on renal and cardiac fibrosis but antifibrotic effects on pulmonary fibrosis.

### VEGF/VEGFR signaling pathway

The VEGF/VEGFR pathway, which comprises six VEGF isoforms (VEGF-A to -E and PlGF) and two receptors (VEGFR1 and VEGFR2), extensively participates in angiogenesis, vasculogenesis, and immunity.^349^ Dysregulation of VEGF signaling can lead to increased vascular permeability, endothelial proliferation, and aberrant angiogenesis, contributing to various diseases, including organ fibrosis.^350^

In chronic liver injury, VEGFA promotes biliary epithelial cell (BEC)-to-hepatocyte conversion and reduces steatosis and fibrosis by targeting KDR receptors on BECs.^351^ In a rat model of myocardial infarction, targeted therapy using VEGF-CC152S, a variant of VEGF-C, enhances cardiac lymphangiogenesis, improves myocardial fluid balance, and mitigates cardiac inflammation and fibrosis by restoring lymphatic transport capacity.^352^ In MASLD and HCC, hepatocyte-derived VEGFA activates HSCs via VEGF-VEGFR signaling in lipid-enriched microenvironments, triggering fibrogenic phenotypic transformation characterized by upregulated TGF-β1/TIMP1.^353^ In IPF, hypoxia-induced HIF-1α activation preferentially upregulates profibrotic VEGF-Axxxa isoforms in lung fibroblasts through altered VEGF-A gene splicing, driving fibronectin expression and collagen deposition.^354^ Interestingly, hyperoxia similarly upregulated VEGF-Axxxa and fibronectin in IPF-derived fibroblasts, further exacerbating fibrogenesis via dysregulated VEGFR1/NRP1 signaling. Despite its profibrotic effects in some contexts, VEGF plays a protective role in IPF. Reduced VEGF levels in the lungs and blood contribute to epithelial injury and fibroblast activation. VEGF overexpression in mice protects against bleomycin-induced lung injury via an endothelial cell-dependent mechanism involving TSP1, highlighting the role of VEGF in maintaining epithelial homeostasis.^355^

### ER stress signaling

The endoplasmic reticulum (ER) functions as a quality-control organelle that regulates protein homeostasis through ER-associated degradation, chaperone-mediated folding, and autophagy. Disruption of proteostasis leads to ER stress, which is characterized by the accumulation of misfolded/unfolded proteins, triggering the unfolded protein response (UPR).^356^ The UPR orchestrates either adaptive restoration of ER homeostasis or maladaptive cell death through three transmembrane sensors: IRE1α, PERK, and ATF6. Mechanistically, misfolded proteins sequester Bip from these sensors, inducing their phosphorylation and subsequent activation.

Accumulating evidence indicates that dysregulated ER stress and maladaptive UPR in fibrogenesis occur via cell death, inflammation, EMT, and myofibroblast transdifferentiation. The progression of MAFLD to liver fibrosis is closely associated with ER stress, which ultimately leads to the activation of liver fibrosis and ECM deposition.^168^ Macrophage-specific XBP1 deficiency attenuates liver fibrosis by shifting M1-to-M2 macrophage polarization and suppressing proinflammatory cytokines.^357^ Specifically, ATF4-mediated ER stress exacerbates fibrosis by inducing EMT in HSCs.^358^ Pulmonary fibrosis is correlated with ER stress in AT2 epithelial cells and macrophages, whereas BiP deficiency in IPF patients potentiates TGF-β signaling and fibrogenesis.^359^ Furthermore, ER stress signaling has been shown to mediate mechanical stretch- or mechanical ventilation-induced extracellular vesicle release, further promoting fibroblast activation during mechanical ventilation-induced pulmonary fibrosis.^360^ Cardiac fibrosis is characterized by elevated phospho-PERK and IRE1α levels, although ATF6 paradoxically suppresses TGF-β-driven fibroblast activation.^361^ Additionally, ER stress induces fibrosis and apoptosis in human kidney proximal tubular cells via autophagy, with PERK inhibition demonstrating selective antifibrotic effects.^362^ While ER stress universally contributes to multiorgan fibrosis, its dichotomous roles (adaptive vs. maladaptive UPR) necessitate therapeutic caution. Future mechanistic studies are warranted to elucidate cell type-specific ER stress responses in the pathogenesis of fibrosis.

## Antifibrotic drugs and clinical trials

The global burden of fibrosis contributes significantly to morbidity and mortality across a spectrum of chronic diseases. However, therapeutic drugs for fibrosis remain remarkably limited. To date, only pirfenidone and nintedanib have been approved specifically for their antifibrotic effects, both for IPF therapy. Pirfenidone has a pleiotropic mechanism of action. Although its precise molecular targets are diverse and not yet fully understood, a cornerstone of its efficacy is the inhibition of the synthesis and activation of TGF-β, thereby suppressing its downstream signaling cascade. In contrast, nintedanib functions as a potent intracellular inhibitor that targets key receptor tyrosine kinases, specifically VEGFR, FGFR, and PDGFR. By blocking these signaling pathways, nintedanib disrupts key processes in IPF pathogenesis, such as the proliferation and migration of lung fibroblasts and their differentiation into matrix-producing myofibroblasts. Notably, both agents can slow the progression of IPF but cannot reverse established fibrosis or confer a definitive survival advantage. A significant advancement was the FDA approval of resmetirom as the first-ever drug for the treatment of MASH. As a liver-directed, selective thyroid hormone receptor-β agonist, it promotes hepatic fatty acid oxidation and reduces lipotoxicity, thereby exerting an indirect antifibrotic effect.^4^ Fortunately, the FDA granted accelerated approval of semaglutide on August 15, 2025, for the treatment of noncirrhotic MASH accompanied by moderate to severe liver fibrosis.^5^ Semaglutide, a GLP-1 receptor agonist, primarily reduces weight and improves glucose tolerance, indirectly exerting antifibrotic effects.

In addition to these approved therapies, numerous antifibrotic drugs that target critical signaling pathways are currently undergoing preclinical investigations or clinical trials. Here, we summarize these drugs on the basis of their specific targets and rank them according to their mechanisms of action within each signaling pathway. Additionally, the drug-based strategies undergoing clinical trials for fibrosis are detailed in Table 3.

**Table 3:** Overview of drug-based strategies in clinical trials for fibrosis

Targets	Mechanism of action	Drug name	Disease	Phase	Trial information	Outcome	Status
TGF-β signaling pathway	Inhibition of TGF-β production	Pirfenidone	IPF	Marketed	NCT00662038	Efficacious and well-tolerated	Completed
			Advanced liver fibrosis	II	NCT04099407	Efficacious and safe	Completed
			Compensated liver cirrhosis	II	NCT06267794	Efficacious and safe	Completed
			HCV-associated cirrhosis	II	NCT02161952	Efficacious	Completed
			Myocardial fibrosis	II	NCT02932566	Reduced myocardial fibrosis	Completed
			FSGS	II	NCT00001959	Improvement in eGFR decline, but no effect on albuminuria	Completed
			DN	I/II	NCT00063583	Unknown	Completed
			DN	III	NCT02689778	Unknown	Completed
			DN	IV	NCT06224790	Unknown	Completed
			CKD	I	NCT04126538	Ongoing	Recruiting
			CKD	II	NCT04258397	Ongoing	Recruiting
		Hydronidone	HBV-associated cirrhosis	III	NCT05115942	Unknown	Completed
			HBV-associated cirrhosis	III	NCT05905172	Ongoing	Recruiting
		HEC585	IPF	II	NCT05060822	Ongoing	Recruiting
			Progressive fibrosing interstitial lung diseases	II	NCT05139719	Ongoing	Recruiting
		TRK-25	IPF	I	NCT03727802	Safe and well-tolerated	Completed
	Neutralization of TGF-β ligand	Fresolimumab	FSGS	II	NCT01665391	Well tolerated, but endpoints were not met	Completed
			IPF	I	NCT00125385	Unknown	Completed
		LY2382770	DN	II	NCT01113801	Lack of efficacy	Terminated
Wnt signaling pathway	Inhibition of β-catenin/CBP interaction	PRI-724	HBV and HCV associated cirrhosis	I/II	NCT03620474	Efficacious	Completed
			PBC	I	NCT04047160	Well tolerated	Completed
	Frizzled internalization modulator	Niclosamide	DN	III	NCT04317430	Reduction in albuminuria	Completed
MAPK signaling pathway	MAPKKK5 inhibitor	Selonsertib	MASH and stage 3 fibrosis	III	NCT03053050	Lack of efficacy	Terminated
			MASH and compensated cirrhosis	III	NCT03053063	Lack of efficacy	Terminated
			Severe alcoholic hepatitis	II	NCT02854631	Lack of efficacy	Completed
			DN	II	NCT04026165	Primary endpoints were met	Completed
	MAPKKK19 inhibitor	MG-S-2525	IPF	I	NCT03650075	Unknown	Completed
	JNK inhibitors	CC-930	IPF	II	NCT01203943	Unsafe	Terminated
		CC-90001	IPF	II	NCT03142191	Unknown	Terminated
			MASH and stage 2/3 fibrosis	II	NCT04048876	Unknown	Terminated
JAK/STAT signaling pathway	JAK inhibitors	Baricitinib	PBC	II	NCT03742973	Unknown	Terminated
			DN	II	NCT01683409	Reduction in albuminuria	Completed
		Jaktinib	IPF	II	NCT04312594	Unknown	Completed
PI3K/AKT signaling pathway	PI3K/mTOR inhibitors	GSK2126458	IPF	I	NCT01725139	Good tolerability	Completed
		HEC-68498	IPF	I	NCT03502902	Unknown	Completed
PPAR signaling pathway	PPARα agonists	Fenofibrate	PBC	II	NCT00575042	Improved serum ALP	Completed
			PBC	III	NCT06755151	Ongoing	Recruiting
		Bezafibrate	PBC	III	NCT01654731	Improved liver stiffness	Completed
		Pemafibrate	MASH	II	NCT03350165	Improved liver stiffness	Completed
			PBC	II	NCT06247735	Ongoing	Active, not recruiting
	PPARγ agonist	Pioglitazone	MASH	IV	NCT00994682	Fibrosis improvement	Completed
			Chronic hepatitis B and diabetes	IV	NCT04584242	Unknown	Unknown
	PPARα/δ agonist	Elafibranor	MASH	III	NCT02704403	Lack of efficacy	Terminated
			PBC	II	NCT03124108	Biochemical improvement	Completed
	PPARα/γ agonist	Saroglitazar	PBC	II	NCT03112681	Improved serum ALP	Completed
			MASH	II	NCT03061721	Fibrosis improvement	Completed
	PPAR α/δ/γ agonist	Lanifibranor	MASH	II	NCT03008070	Fibrosis improvement	Completed
			MASH	III	NCT04849728	Ongoing	Recruiting
Integrin signaling pathway	Anti-αVβ3 integrin antibody	VPI-2690B	DN	II	NCT02251067	Unknown	Completed
	Anti-αVβ6 integrin antibody	BG00011	IPF	II	NCT03573505	Unsafe	Terminated
	Antagonist of αvβ6 integrin	GSK3008348	IPF	I	NCT02612051	Well-tolerated	Completed
	Antagonist of αvβ1and αvβ6 integrins	PLN-74809	IPF	II	NCT04396756	Efficacious	Completed
	Antagonist of αvβ1and αvβ6 integrins	PLN-74809	IPF	II	NCT05621252	Efficacious	Completed
			IPF	II	NCT04072315	Efficacious	Completed
			IPF	II	NCT06097260	Ongoing	Active, not recruiting
	Antagonist of αvβ1, αvβ3, and αvβ6 integrins	IDL-2965	IPF	I	NCT03949530	Unknown	Terminated
RhoA/ROCK signaling pathway	Selective ROCK2 inhibitor	Belumosudil	IPF	II	NCT02688647	Improved lung function	Completed
		Zelasudil	IPF	I	NCT04931147	Unknown	Completed
			IPF	II	NCT05570058	Ongoing	Active, not recruiting
FGF/FGFR signaling pathway	FGF19 analog	Aldafermin	MASH and stage 2/3 fibrosis	II	NCT02443116	Fibrosis improvement	Completed
			MASH and stage 2/3 fibrosis	II	NCT03912532	Fibrosis improvement	Completed
			MASH and compensated cirrhosis	II	NCT04210245	Fibrosis improvement	Completed
	FGF21 analog	Pegbelfermin	MASH and stage 1-3 fibrosis	II	NCT02413372	Fibrosis improvement	Completed
			MASH and stage 3 fibrosis	II	NCT03486899	Primary endpoints were not met	Completed
			MASH and compensated cirrhosis	II	NCT03486912	Primary endpoints were not met	Completed
		Pegozafermin	MASH and stage 2/3 fibrosis	II	NCT04929483	Fibrosis improvement	Completed
			MASH and stage 2/3 fibrosis	III	NCT06318169	Ongoing	Recruiting
			MASH and compensated cirrhosis	III	NCT06419374	Ongoing	Recruiting
		Efruxifermin	MASH and stage 2/3 fibrosis	II	NCT04767529	Fibrosis improvement	Completed
			MASH and compensated cirrhosis	II	NCT03976401	Fibrosis improvement	Completed
			MASH and stage 2/3 fibrosis	III	NCT06215716	Ongoing	Recruiting
			MASH and compensated cirrhosis	III	NCT06528314	Ongoing	Recruiting
RTK signaling pathway	RTK inhibitors	Nintedanib	IPF	III	NCT02999178	Efficacious	Completed
			IPF	III	NCT01335464	Efficacious and well-tolerated	Completed
			IPF	III	NCT01335477	Efficacious and well-tolerated	Completed
			IPF	IV	NCT02598193	Good tolerability	Completed
		ZSP1603	IPF	I and II	NCT05119972	Unknown	Completed
		Anlotinib	IPF	II and III	NCT05828953	Ongoing	Recruiting

*IPF* idiopathic pulmonary fibrosis, *HCV* hepatitis C virus, *FSGS* focal segmental glomerulosclerosis, *eGFR* estimated glomerular filtration rate, *DN* diabetic nephropathy, *CKD* chronic kidney disease, *HBV* hepatitis B virus, *MASH* metabolic dysfunction-associated steatohepatitis, *PBC* primary biliary cholangitis, *TGF β* transforming growth factor β, *CBP* cyclic AMP response-element binding protein, *MAPK* mitogen-activated protein kinase, *JNK* c-Jun amino terminal kinase, *JAK* Janus kinase, *PI3K* phosphatidylinositol-3-kinase, *mTOR* mammalian target of rapamycin, *PPAR* peroxisome proliferator-activated receptor, *ROCK* Rho-associated coiled-coil kinase, *FGF* fibroblast growth factor, *FGFR* fibroblast growth factor receptor, *RTK* receptor tyrosine kinase

### Anti-fibrotic drugs targeting the TGF-β signaling pathway

#### Inhibition of TGF-β production

Pirfenidone is an orally bioavailable small molecule that exerts antifibrotic effects by inhibiting the TGF-β signaling pathway. Pirfenidone, which was approved by the FDA in 2014 for IPF, is under clinical investigation for various other fibrotic diseases, including liver fibrosis, myocardial fibrosis, focal segmental glomerulosclerosis (FSGS), and diabetic nephropathy (DN). A phase II trial (NCT04099407) demonstrated significant fibrosis reduction in 35% of chronic liver disease patients treated with pirfenidone.^363^ Additional studies in patients with compensated cirrhosis (NCT06267794)^364^ and HCV-associated cirrhosis (NCT02161952)^365^ corroborated its antifibrotic efficacy. Pirfenidone also improved myocardial fibrosis in heart failure patients with preserved ejection fraction (NCT02932566)^365^ and slowed the decrease in the glomerular filtration rate in FSGS patients (NCT00001959) without affecting proteinuria.^366^ Ongoing trials (NCT04126538 and NCT04258397) are further evaluating its renoprotective effects, but results from DN studies (NCT00063583, NCT02689778, and NCT06224790) are lacking.

Hydronidone, a pirfenidone derivative, has shown efficacy in reversing HBV-associated liver fibrosis after 52 weeks of treatment.^367^ A completed phase III trial (NCT05115942) assessed its 270 mg/day dosage, although the results remain unpublished. A phase IIIb trial (NCT05905172) is currently evaluating long-term outcomes and antifibrotic durability.

HEC-585 (Yinfenidone), a metabolically stable pyrimidone analog of pirfenidone, exhibits enhanced pharmacokinetics and antifibrotic activity in preclinical pulmonary fibrosis models.^368^ Two ongoing clinical trials (NCT05060822 and NCT05139719) are assessing its therapeutic efficacy in pulmonary fibrosis.

TRK-250, a single-stranded oligonucleotide conjugated with two proline linkers, generates siRNAs targeting human TGFβ1 mRNA, reducing TGF-β1 expression and collagen production in animal models. A phase I study (NCT03727802) confirmed its safety and tolerability in IPF patients.^369^ However, further clinical trials are needed to validate the antifibrotic efficacy of TRK-250 in humans.

#### Neutralization of the TGF-β ligand

Fresolimumab, a high-affinity human anti-pan-TGF-β monoclonal antibody, targets all three TGF-β isoforms. Although well tolerated in FSGS patients,^370^ Fresolimumab failed to meet primary efficacy endpoints for proteinuria reduction in a phase II trial (NCT01665391).^371^ A phase I trial involving patients with IPF (NCT00125385) has been completed, but outcomes have yet to be reported.

LY2382770, a humanized TGF-β1-neutralizing monoclonal antibody, was evaluated in a phase II trial for DN patients (NCT11133801). However, it failed to significantly improve the mitigation of progressive renal fibrosis and preservation of renal function.^372^

### Anti-fibrotic drugs targeting the Wnt signaling pathway

#### Inhibition of the β-catenin/CBP interaction

ICG-001 can specifically bind to CREB-binding protein (CBP), disrupting β-catenin/CBP complex formation. Preclinical studies have demonstrated its antifibrotic efficacy in renal, cardiac, liver, and lung fibrosis models, supporting further clinical development.^373^

PRI-724 (OP-724), a second-generation β-catenin/CBP inhibitor, has therapeutic potential in liver fibrosis. Phase I/II trials in patients with HBV/HCV-induced cirrhosis (NCT03620474) revealed improved liver stiffness and fibrosis-related biomarkers after 12 weeks of intravenous administration.^374^ A phase I study of primary biliary cholangitis (PBC) (NCT04047160) confirmed its safety, although its antifibrotic efficacy warrants further evaluation.

#### Frizzled internalization modulator

Niclosamide inhibits Wnt/β-catenin signaling by promoting Frizzled-1 endocytosis and downregulating Dishevelled-2.^375^ In a randomized trial (NCT04317430), adjunct niclosamide therapy reduced albuminuria in DN patients receiving ACE inhibitors. Additional studies are needed to assess its antifibrotic effects in humans.

### Antifibrotic drugs targeting the MAPK signaling pathway

#### MAPKKK inhibitors

Selonsertib, a highly selective small-molecule inhibitor of MAPKKK5, was evaluated in two phase III clinical trials for MASH with different stages of fibrosis (NCT03053050 and NCT03053063) but showed no significant efficacy.^376,377^ A phase II trial in severe alcoholic hepatitis patients (NCT02854631) also failed to meet its primary endpoint. However, a phase II study in DN patients (NCT04026165) achieved its primary endpoint by slowing the decrease in the eGFR,^378^ implying a potential therapeutic benefit.

MG-S-2525, a MAPKKK19 inhibitor, has completed a phase I trial for IPF (NCT0365007), although the results have not yet been released.

#### JNK inhibitors

CC-930 is a first-generation JNK2-biased inhibitor that was evaluated in a phase II study in patients with IPF (NCT01203943). However, its development was discontinued because of hepatotoxicity.

CC-90001, a second-generation JNK-selective inhibitor with improved safety,^379^ was investigated for the treatment of pulmonary (NCT0314219) and hepatic fibrosis (NCT04048876). However, both trials were prematurely terminated because of business issues.

### Antifibrotic drugs targeting the Hippo/YAP signaling pathway

Verteporfin, a YAP inhibitor, disrupts YAP-TEAD complex formation and has demonstrated antifibrotic effects in UUO-induced renal fibrosis, CCl_4_-induced liver fibrosis, and bleomycin-induced lung fibrosis models.^380,381^ Furthermore, verteporfin treatment has been shown to improve cardiac function and reduce fibrosis in mice following MI. Clinical studies are warranted to evaluate its therapeutic potential in fibrotic diseases.

### Antifibrotic drugs targeting the JAK/STAT signaling pathway

#### JAK inhibitors

Ruxolitinib, a selective JAK1/JAK2 inhibitor, was the first FDA-approved drug for myelofibrosis and has demonstrated efficacy for other fibrotic diseases in preclinical studies. A case report described marked improvement in a patient with IPF and COVID-19 following ruxolitinib treatment.^382^ Additionally, ruxolitinib attenuates cardiac fibrosis by suppressing fibroblast activation and inflammatory cell activity.^383^ Preclinical studies further support its efficacy in reducing CCl_4_- or TAA-induced liver fibrosis and UUO-induced renal fibrosis,^384^ warranting further clinical validation.

Baricitinib is a selective JAK1/JAK2 inhibitor that is clinically used for rheumatoid arthritis. While a trial in PBC patients (NCT03742973) was terminated owing to futility at enrollment, a phase II study in DN patients (NCT01683409) demonstrated reduced albuminuria and inflammatory marker levels.^385^ However, its direct antifibrotic effects against DN require further investigation.

Jaktinib is a novel pan-JAK inhibitor that has potential in various inflammatory and fibrotic diseases.^386^ A phase II study in IPF (NCT04312594) has been completed, but the results are pending.

#### STAT inhibitors

S3I-201 is a novel specific STAT3 inhibitor that preferentially inhibits STAT3 DNA-binding activity while diminishing STAT3 tyrosine phosphorylation. In a UUO-induced renal fibrosis mouse model, S3I-201 suppressed fibroblast activation and profibrotic marker expression.^387^ It also reduced left atrial fibrosis with myocardial infarction and attenuated HSC activation in a CCl_4_-induced liver fibrosis model.^388^

### Anti-fibrotic drugs targeting the PI3K/AKT signaling pathway

#### PI3K/mTOR inhibitors

GSK2126458 (Omipalisib), a potent and highly selective pyridylsulfonamide inhibitor of PI3K/mTOR, significantly inhibits AKT phosphorylation, attenuates fibroblast proliferation, and reduces TGF-β-induced collagen deposition in lung tissues.^389^ A phase I trial (NCT01725139) demonstrated favorable pharmacokinetics and tolerability in IPF patients, supporting further efficacy evaluation.^390^

HEC-68498 is a potent and highly selective inhibitor of PI3K/mTOR that was developed by Guangdong Dongyangguang Pharmaceutical Co., Ltd. HEC-68498 is currently undergoing phase I clinical trials for IPF in China (NCT03502902).

### Antifibrotic drugs targeting the PPAR signaling pathway

#### PPARα agonists

Fenofibrate is an agonist of PPARα approved for hypercholesterolemia treatment. In a phase II trial in patients with PBC (NCT00575042), combination therapy with fenofibrate and ursodeoxycholic acid for 48 weeks significantly improved fibrosis, as indicated by lower serum alkaline phosphatase (ALP) activity.^391^ An ongoing study (NCT06755151) is evaluating its long-term safety in PBC.

Bezafibrate is a PPARα agonist developed for the treatment of mixed hyperlipidemia. In a phase III study for PBC (NCT01654731), bezafibrate combined with ursodeoxycholic acid significantly improved fibrosis biomarkers and symptoms,^392^ highlighting its therapeutic potential for cholestatic liver fibrosis.

Pemafibrate is a novel selective PPARα modulator that can ameliorate hepatic steatosis, inflammation, and fibrogenesis in high-fat diet-fed murine models.^393^ A phase II trial (NCT03350165) demonstrated its efficacy in improving dyslipidemia and reducing liver stiffness in MASH patients.^394^ An ongoing phase II trial (NCT06247735) is investigating its therapeutic efficacy and safety in PBC patients.

#### PPARγ agonists

Pioglitazone, a PPARγ agonist, significantly attenuated liver fibrosis and enhanced insulin sensitivity in MASH patients with type 2 diabetes, with more pronounced effects than in prediabetic individuals in a phase IV clinical trial (NCT00994682).^395^ A subsequent phase IV clinical trial (NCT04584242) is assessing its efficacy in chronic hepatitis B and diabetes patients.

#### PPARα/δ agonists

Elafibranor is a dual PPARα/δ agonist that has demonstrated hepatoprotective effects via the modulation of lipid metabolism and fibrotic signaling in preclinical studies. While a phase III MASH trial (NCT02704403) failed to meet endpoints, elafibranor successfully decreased serum ALP in PBC patients during a phase II trial (NCT03124108).^396^

#### PPARα/γ agonists

Saroglitazar is a PPARα/γ dual agonist that can regulate glucose metabolism and improve insulin resistance. Phase II trials in PBC (NCT03112681)^397^ and MASH (NCT03061721)^398^ demonstrated its potential to mitigate liver fibrosis.

#### PARα/δ/γ agonists

Lanifibranor is a PPAR α/δ/γ triple agonist that has robust antifibrotic effects in animal models.^399^ A phase II trial (NCT03008070) confirmed its ability to induce MASH resolution and fibrosis regression, promoting an ongoing phase III study (NCT04849728) in MASH patients with stage 2--3 fibrosis.^400^

### Anti-fibrotic drugs targeting the Notch signaling pathway

#### γ secretase inhibitors

DAPT is a potent and specific γ-secretase inhibitor (GSI) that inhibits the release of the Notch intracellular domain. Preclinical studies have demonstrated its efficacy in attenuating fibrosis in CCl_4_-induced liver fibrosis, DN, and lung fibrosis models.^401^

RO4929097, another selective GSI, attenuated UUO-induced renal fibrosis and liver fibrosis in preclinical studies.^402^

Crenigacestat, a selective GSI, reduces liver fibrosis in cholangiocarcinoma xenografts by suppressing TGF-β1/SMAD2 signaling and ECM production.^271^

Avagacestat is another selective GSI that inhibits the TGFβ-induced activation and contractility of HSCs, mitigating CCl_4_-induced liver fibrosis.^403^

### Anti-fibrotic drugs targeting the integrin signaling pathway

#### Anti-integrin antibodies

VPI-2690B is a monoclonal antibody that specifically targets integrin αVβ3, which plays a crucial role in TGF-β signaling.^404^ A phase II trial (NCT02251067) conducted to evaluate the safety and efficacy of VPI-2690B in DN patients remains unpublished.

BG00011 (STX-100) is a first-in-class humanized anti-αvβ6 monoclonal antibody^405^ that inhibits the activation of latent TGF-β. Phase II trials for IPF (NCT03573505) were discontinued because of safety concerns.

#### Integrin antagonists

GSK3008348, an inhaled αvβ6 integrin inhibitor, has been shown to prolong TGF-β inhibition in bleomycin-induced lung fibrosis.^406^ Phase I data (NCT02612051) support its safety in IPF.

PLN-74809 (bexotegras) is an oral small-molecule αvβ1/αvβ6 dual inhibitor with promising phase I/II results in IPF, improving lung functions and biomarkers (NCT04396756, NCT05621252, and NCT04072315).^407,408^ An ongoing phase II trial (NCT06097260) further evaluated its efficacy and safety in IPF patients.

IDL-2965 is an oral panαv integrin antagonist that has shown therapeutic potential for IPF and MASH.^409^ Unfortunately, a phase I trial (NCT03949530) in both healthy subjects and IPF patients was halted because of COVID-19 and preclinical challenges.

### Anti-fibrotic drugs targeting the cGAS–STING signaling pathway

#### cGAS inhibitors

RU.521 can competitively occupy the catalytic site of cGAS,^410^ thereby impairing its binding to ATP/GTP and suppressing 2’,3’-cGAMP synthesis. In a preclinical model of obstructive nephropathy, RU.521 attenuated cGAS-STING activation, significantly reducing inflammation and fibrosis in the kidney.^411^

#### STING inhibitors

H-151 can inhibit palmitoylation-dependent STING aggregation by covalently modifying STING at the Cys91 residue. It effectively suppresses STING-induced inflammatory cytokine production and fibrosis markers in postmyocardial infarction hearts, thereby mitigating cardiac hypertrophy.^412^

#### TBK1 inhibitors

Amlexanox, a dual TBK1/IKKε inhibitor, has been shown to reduce hepatic inflammation, metabolic dysfunction, and steatosis in MASH mouse models.^413^ It also alleviated renal fibrosis and restored kidney function in UUO-induced CKD model mice.^414^

### Antifibrotic drugs targeting the RhoA/ROCK signaling pathway

Belumosudil is an FDA-approved oral ROCK2 inhibitor for chronic graft-versus-host disease.^415^ In a clinical trial for IPF patients (NCT02688647), Belumosudil reduced the median decline in total forced vital capacity by 73% compared with standard care, supporting its further development.

Zelasudil (RXC007) is a highly potent, selective, and orally active ROCK2 inhibitor that has demonstrated strong efficacy in preclinical lung fibrosis models.^416^ Clinical trials for IPF (NCT05570058) and safety studies (NCT04931147) are ongoing, with no results posted.

### Anti-fibrotic drugs targeting the FGF/FGFR signaling pathway

#### FGF 19 analogs

Aldafermin, also referred to as NGM282/M70, is an engineered FGF19 analog that selectively activates the FGFR4/KLB coreceptor complex. Aldafermin improved liver inflammation and fibrosis in MASH patients across multiple trials (NCT02443116, NCT03912532).^417–419^ Aldafermin also showed efficacy in MASH patients with compensated cirrhosis (NCT04210245).^420^

#### FGF 21 analogs

Pegbelfermin (BMS-986036) is a PEGylated analog of human FGF21 that was engineered to extend the half-life of endogenous FGF21.^421^ In a 16-week phase II study in MASH patients and stage 1-3 fibrosis patients (NCT02413372), pegbelfermin treatment reduced hepatic fat and improved metabolic factors and biomarkers of hepatic injury and fibrosis.^422^ However, the primary endpoint in advanced fibrosis was missed in a randomized phase IIb study (NCT03486899, NCT03486912).^423,424^

Pegozafermin (BIO89-100) is a novel glycoPEGylated FGF21 analog with an extended circulating half-life of 55-100 h.^421^ In a phase IIb trial for MASH and stage 2/3 fibrosis (NCT04929483), pegozafermin improved liver fibrosis with biweekly dosing,^425^ advancing to phase III development (NCT06318169, NCT06419374).

Efruxifermin is a long-acting Fc-FGF21 fusion protein that has demonstrated safety and metabolic and fibrosis improvements in multiple phase II MASH trials with different stages of liver fibrosis and cirrhosis (NCT04767529, NCT03976401).^426,427^ Ongoing phase III trials are expected to provide more definitive conclusions on the therapeutic efficacy of Efruxifermin for MASH and liver fibrosis (NCT06215716, NCT06528314).

### Antifibrotic drugs targeting the RTK signaling pathway

#### RTK inhibitors

Nintedanib (BIBF1120) is an orally potent small-molecule RTK inhibitor that targets FGFR, PDGFR, and VEGFR, ultimately inhibiting fibroblast proliferation and differentiation.^428^ Clinical trials (NCT02999178, NCT01335464, NCT01335477, and NCT02598193) have confirmed its efficacy and tolerability in IPF patients, leading to its approval as a soft gelatin capsule formulation.

ZSP1603 is a novel RTK inhibitor that targets FGFR, PDGFR, and VEGFR, which can attenuate TGF-β1, collagen I, and α-SMA upregulation, demonstrating antifibrotic effects in bleomycin-induced pulmonary fibrosis models.^17^ A phase I/II trial (NCT05119972) has been completed, although the results remain unpublished.

Anlotinib is a novel RTK inhibitor that shares the same targets as nintedanib and reduces fibrosis in liver, lung, and kidney models. Phase II/III trials (NCT05828953) are ongoing for IPF.

### Antifibrotic drugs targeting ER stress signaling

#### IRE1α inhibitors

KIRA8 is a nanomolar-potent, monoselective small-molecule IRE1α modulator. A preclinical study demonstrated that KIRA8 significantly ameliorated pulmonary fibrosis in bleomycin-treated mice by inhibiting the IRE1α‒XBP1 pathway.^429^

STF-083010 is a specific IRE1α inhibitor that mitigates thioacetamide-induced liver injury and fibrosis in mice.^430^ By suppressing IRE1α RNase activity, STF-083010 also impedes steatosis-to-MASH progression and improves glucose tolerance under chronic ER stress.^431^

4μ8C is an inhibitor of IRE1α that has demonstrated therapeutic efficacy against hepatic fibrosis in alcohol- and CCl_4_-induced liver fibrosis models.^432^ In addition, it attenuated M1 macrophage polarization and renal fibrosis in a UUO mouse model.^433^

#### ER chaperones

4-Phenylbutyric acid (4‑PBA) is an FDA-approved aromatic fatty acid analog for the treatment of urea cycle disorders and sickle cell disease. As an ER chaperone, 4-PBA inhibits ER stress and UPR signaling, thereby preventing fibrogenesis in various preclinical fibrosis models.^434^

## Future directions

### Multiomics integration

The rapid advancement of high-throughput omics has deepened our understanding of fibrotic pathogenesis across organs, revealing spatiotemporal molecular and cellular dynamics. While single-cell studies have revealed cellular heterogeneity during remodeling, reliance on single-omics data has limited fibrosis characterization to the transcriptomic or epigenomic level^435^. Future research should integrate multimodal single-cell omics data—encompassing genomics (genetic variation), transcriptomics (gene expression profiles), epigenomics (chromatin accessibility and regulatory modifications), proteomics (protein interactions and posttranslational modifications), metabolomics (metabolic reprogramming), and spatial transcriptomics/proteomics—to comprehensively annotate cellular states, ontogeny, and function throughout fibrosis progression in humans.^2^ Although the pathogenesis of fibrosis varies across organs, recent single-cell studies have identified specific cell subsets, such as FAP^+^ fibroblasts, PLVAP^+^ endothelial cells, and SPP1^+^ macrophages, that play roles in regulating fibrosis across multiple organs (Table 4). However, the mechanisms by which the precursor cells of these subpopulations respond to microenvironmental stimuli and differentiate into either profibrotic or antifibrotic phenotypes remain poorly understood. Critical questions include whether key transcription factors govern this phenotypic transition or if it is driven by metabolic reprogramming. Additionally, it is unclear how these cells, in turn, reshape their niche through the secretion of effector molecules to coordinate a fibrotic response with neighboring cells, thereby collectively contributing to the fibrotic process. Addressing these complex questions necessitates integrated multiomic analyses across multiple organs to identify potential pantissue therapeutic targets.

**Table 4:** Summary of the major cell subsets involved in the pathogenesis of organ fibrosis

Cell type	Phenotype	Organ	Advantage	Disadvantage	Reference
Fibroblast	CTHRC1^+^	Lung, heart	NA	High migration ability, highly activated phenotype; producing pathologic ECM	^500,501^
	CD248^+^	Kidney, lung, heart, liver	Pro-resolving in lung	Promoting T-cell infiltration and retention; negatively correlated with renal function.	^502–504^
	FAP^+^	Kidney, lung, heart, liver	NA	Promoting both macrophage and HSC profibrogenic activity; producing pathologic ECM	^494,505–507^
	POSTN^+^	Lung, heart	NA	Associated with pathological extracellular matrix remodeling	^481,508^
	PDGFRB^+^	Kidney, liver	NA	Pathological proliferation; inducing progressive organ fibrosis and failure	^509,510^
Endothelial cell	PLVAP^+^	Kidney, lung, heart, liver	NA	Promoting the transmigration of leucocytes; endothelial leakiness	^85,140,219,511^
	ACKR1^+^	Lung, heart, liver	NA	Promoting inflammation; regulating leucocyte extravasation and ECM deposition	^141,219,512^
Macrophage	CX3CR1^+^	Kidney, lung, heart	NA	Promoting the proliferation of fibroblasts and tissue fibrosis	^99,154^
	SPP1^+^	Kidney, lung, heart, liver	Improving liver steatosis by enhancing FAO in hepatocytes	Promoting myofibroblast activation, collagen deposition, and scar formation	^153,513–515^
	CCR2^+^	Lung, heart, liver	NA	Localized relative to fibrotic regions; releasing MCP-1 to promote inflammation	^516–518^
	TREM2^+^	Kidney, lung, liver	Facilitating efferocytosis of lipid-laden apoptotic hepatocytes; promoting collagen degradation	Pro-fibrotic phenotype in idiopathic pulmonary fibrosis	^307,519,520^
Neutrophil	Siglec-F^+^	Kidney, lung, heart	NA	Promoting the activation of fibroblasts to instigate excessive fibrosis; releasing toxic NETs; higher expression of profibrotic inflammatory cytokines	^521,522^

*CTHRC1* collagen triple helix repeat containing 1, *CD248* also known as endosialin or tumor endothelial marker-1, *FAP* fibroblast activation protein alpha, *POSTN* periostin, *PDGFRB* platelet-derived growth factor receptor beta, *PLVAP* plasmalemma vesicle-associated protein, *ACKR1* atypical chemokine receptor 1, *CX3CR1* chemokine (C-X3-C motif) receptor 1, *SPP1* secreted phosphoprotein 1, *CCR2* C-C motif chemokine receptor 2, *TREM2* triggering receptor expressed on myeloid cells 2, *Siglec-F* sialic acid binding Ig-like lectin F, *FAO* fatty acid oxidation, *NETs* neutrophil extracellular traps, *ECM* extracellular matrix, *MCP-1* monocyte chemoattractant protein-1

Translating insights gained from multiomics integration into effective antifibrotic therapies requires overcoming several challenges. First, fundamental differences in etiology and disease progression exist between the induction methods used in various animal disease models and long-term, multifactorial, naturally occurring diseases in humans, which poses significant challenges for identifying viable therapeutic targets for fibrosis. Furthermore, owing to interspecies differences in physiological structure, immune response dynamics, metabolic rates, and gene expression profiles, the therapeutic efficacy observed in murine models often fails to translate consistently to clinical settings. Given that fibrosis is a complex and dynamic process influenced by multiple factors, targeting a single mechanism is unlikely to fully prevent or reverse the disease. Therefore, to address these challenges, the application of human-relevant models, such as humanized mice, precision-cut tissue slices, and 3D organoids, may better recapitulate fibrotic pathology. Integrating these models with drug-seq and AI-driven screening has the potential to accelerate candidate drug discovery, which can then be validated in both animal and human 3D systems to expedite clinical translation. Moreover, future antifibrotic strategies are anticipated to involve multitarget interventions and necessitate adaptation throughout the treatment course on the basis of individual patient biomarker profiles, thereby advancing personalized precision medicine to significantly improve therapeutic outcomes.

### Chimeric antigen receptor (CAR) therapy

Chimeric antigen receptor T-cell (CAR-T) therapy, a breakthrough in oncology, reprograms T cells to target specific pathologies^436^. Recently, interest in the application of CAR-T-cell therapy for the treatment of fibrotic diseases has increased, reflecting the field’s ongoing expansion into new areas.^436,437^ For example, fibroblast activation protein (FAP)-targeted CAR-T cells significantly attenuate cardiac fibrosis in AngII/PE-stimulated mice. Furthermore, CAR-T cells that target urokinase plasminogen activator receptor (uPAR), which is highly expressed in senescent and activated HSCs, markedly ameliorate liver fibrosis in mice.^438^ In addition to CAR-T cells, FAP-targeted chimeric antigen receptor macrophages (CAR-Ms) demonstrate significantly enhanced phagocytic capacity, improved efferocytosis, and potent anticardiac fibrosis capabilities.^439,440^ Similarly, uPAR-targeted CAR-Ms can remodel the immune microenvironment, reduce fibroblast numbers, and attenuate hepatic fibrosis.^441^ In addition to the use of CAR-T and CAR-M therapies to target FAP-positive or uPAR-positive activated fibroblasts to reduce excessive ECM deposition, PDGFRβ has been identified as a marker expressed across multiple profibrotic stromal cell populations, including pericytes, fibroblasts, and myofibroblasts.^442^ Notably, PDGFRβ-targeted CAR-T-cell therapy has demonstrated potent antifibrotic activity in mouse models of chronic kidney disease.^442^ These findings highlight the considerable potential of both CAR-M and CAR-T-cell therapies in the treatment of fibrosis-related diseases. Furthermore, target cells are not confined to fibroblasts alone; the clearance of pathogenic pericytes and endothelial cells may also represent a promising direction for future investigations. With respect to the development of CAR-based therapies, the application of CAR-NK and CAR-NKT cells remains an emerging field worthy of further exploration.

Despite preclinical success, several hurdles must be addressed before clinical application. First, the conventional ex vivo manufacturing process for CAR-T cells, which involves blood draws, external genetic modifications, and subsequent reinfusion, is costly, time-consuming, and logistically complex. Second, genetically modified CAR-T cells risk cytokine release syndrome (CRS) and immune dysregulation. Emerging LNP-mRNA-based in vivo CAR-T/M technology enables transient CAR expression, reducing off-target risks.^30,443^ The transient expression of CARs mediated by mRNA–LNP delivery typically persists for only a few days. This feature allows the technology to adapt to the dynamic and heterogeneous microenvironment of fibrosis, enabling adjustable dosing strategies in accordance with disease progression. Furthermore, a combinatorial administration approach can be employed using LNPs loaded with mRNAs encoding CARs that target different antigens. This strategy permits simultaneous targeting of distinct cell populations and the modulation of multiple fibrotic pathways. However, challenges remain within LNP–mRNA therapy, notably its potential to trigger innate immune responses. Further investigations into modifications of LNP components and the mRNA itself are warranted. Highly selective markers for profibrotic cells are essential to minimize off-target toxicity. If repeated dosing is needed, the optimal timing must be judiciously selected to avoid adverse consequences stemming from excessive immune activation.

### Artificial intelligence (AI) technology

Even with significant progress in fibrosis research, challenges persist in early diagnosis, mechanistic understanding, and therapeutic reversal. AI technology is recognized as having immense potential, providing revolutionary tools for the early diagnosis of fibrotic diseases, mechanistic dissection, drug development, and personalized therapy. Early diagnostic limitations include subjective interpretation of histopathology/imaging (e.g., CT/MRI and ultrasound), high interobserver variability in semiquantitative scoring, and insufficient sensitivity in grading systems. Several AI-powered digital pathology platforms currently under development for MASH enable quantitative analysis of histopathological features, such as steatosis, inflammation, hepatocellular ballooning, and fibrosis, in patients. These platforms also capture cellular characteristics, including the count and density of various hepatocyte and immune cell types, as well as the spatial relationships among these features.^444^ They assist pathologists in grading and staging liver biopsies with accuracy and reproducibility, and importantly, they provide continuous metrics for assessing treatment-induced changes. However, these AI-powered digital pathology platforms still lack large-scale, multicenter validation. Additionally, it remains uncertain whether variability in biopsy quality may introduce bias in analyses based on these tools. Even though AI-powered digital pathology using routinely stained slides offers broader feasibility than algorithms based on second harmonic generation (SHG) or two-photon excitation fluorescence (TPEF) microscopy do, a cost-effectiveness analysis of the technology should also be taken into consideration.^445^

AI has demonstrated remarkable efficacy in identifying novel fibrotic targets and facilitating drug development. Researchers have employed a comprehensive set of multiomics data and clinical datasets related to tissue fibrosis to train the target discovery engine within the PandaOmics platform.^446^ This platform identified TNIK as a novel target implicated in driving multiple fibrosis-associated pathways (e.g., the WNT, TGF-β, Hippo, and JNK pathways). They subsequently utilized the generative chemistry engine Chemistry42, which is part of Pharma. AI platforms design innovative molecular structures via structure-based drug design strategies to achieve the desired properties. This process yielded the safe, specific, and highly potent TNIK inhibitor INS018_055, which demonstrated good safety, tolerability, and pharmacokinetic characteristics in a phase 1 clinical trial.^446^ This suggests that training the target discovery engine by inputting multiomics datasets from multiple organs into an AI coscientist may enable the platform to leverage advanced reasoning for synthesizing extensive knowledge from the literature. Such integration could facilitate the generation of novel hypotheses, thereby accelerating the identification of potential antifibrotic therapeutic targets. In addition, AI can also utilize large-scale biomedical datasets to rediscover new therapeutic uses for approved drugs. For example, via machine learning, the potential anti-tubulointerstitial fibrosis effect of the reusable drug lubiprostone was predicted.^447^ However, AI still needs more exploration and data support in optimizing trial designs, predicting patient responses, and thereby improving the efficiency and success rate of trials.^448^

## Conclusions

Fibrosis, a common pathological feature of chronic inflammatory diseases, causes organ dysfunction, severely impacts health, and burdens society. Although approved drugs exist for pulmonary fibrosis and MASH and many candidates are in clinical trials, effective therapies to reverse fibrosis are lacking. Owing to differences in risk factors, injurious stimuli, spatial location, structure, function, and cellular composition between tissues, the pathogenesis of fibrosis varies across organs. Nevertheless, significant commonalities exist, including key signaling pathways governing core processes such as the transdifferentiation and activation of myofibroblasts, loss of parenchymal cell identity, endothelial cell dysfunction, and immune microenvironment remodeling. Future research should leverage cross-organ, integrated multiomics analyses to investigate in depth the phenotypic and functional shifts occurring in various cell types across temporal and spatial dimensions, along with alterations in intercellular communication networks. This will facilitate the mapping of dynamic atlases for individual organs and even multiple organs, aiding in the identification of universal therapeutic targets for fibrosis. The utilization of emerging technologies, including organoids, organs-on-chips, and AI, will further propel stages such as drug design, screening, and efficacy prediction. This progress holds the promise of ushering in a new era of effectively targeted therapies capable of reversing fibrotic diseases.
